# Pattern-mixture model in network meta-analysis of binary missing outcome data: one-stage or two-stage approach?

**DOI:** 10.1186/s12874-020-01205-6

**Published:** 2021-01-07

**Authors:** Loukia M. Spineli, Katerina Papadimitropoulou, Chrysostomos Kalyvas

**Affiliations:** 1 Midwifery Research and Education Unit (OE 9210), Hannover Medical School, Carl-Neuberg-Straße 1, 30625 Hannover, Germany; 2 Clinical Epidemiology, Leiden University Medical Center, Leiden, The Netherlands; 3 Data Science and Biometrics, Danone Nutricia Research, Utrecht, The Netherlands; 4 Biostatistics and Research Decision Sciences, MSD Europe Inc, Brussels, Belgium

**Keywords:** Network meta-analysis, Missing outcome data, Pattern-mixture model, Bayesian methods, One-stage approach, Two-stage approach, Simulation study

## Abstract

**Background:**

Trials with binary outcomes can be synthesised using within-trial exact likelihood or approximate normal likelihood in one-stage or two-stage approaches, respectively. The performance of the one-stage and the two-stage approaches has been documented extensively in the literature. However, little is known about how these approaches behave in the presence of missing outcome data (MOD), which are ubiquitous in clinical trials. In this work, we compare the one-stage versus two-stage approach via a pattern-mixture model in the network meta-analysis using Bayesian methods to handle MOD appropriately.

**Methods:**

We used 29 published networks to empirically compare the two approaches concerning the relative treatment effects of several competing interventions and the between-trial variance (*τ*^2^), while considering the extent and level of balance of MOD in the included trials. We additionally conducted a simulation study to compare the competing approaches regarding the bias and width of the 95% credible interval of the (summary) log odds ratios (OR) and *τ*^2^ in the presence of moderate and large MOD.

**Results:**

The empirical study did not reveal any systematic bias between the compared approaches regarding the log OR, but showed systematically larger uncertainty around the log OR under the one-stage approach for networks with at least one small trial or low event risk and moderate MOD. For these networks, the simulation study revealed that the bias in log OR for comparisons with the reference intervention in the network was relatively higher in the two-stage approach. Contrariwise, the bias in log OR for the remaining comparisons was relatively higher in the one-stage approach. Overall, bias increased for large MOD. For these networks, the empirical results revealed slightly higher *τ*^2^ estimates under the one-stage approach irrespective of the extent of MOD. The one-stage approach also led to less precise log OR and *τ*^2^ when compared with the two-stage approach for large MOD.

**Conclusions:**

Due to considerable bias in the log ORs overall, especially for large MOD, none of the competing approaches was superior. Until a more competent model is developed, the researchers may prefer the one-stage approach to handle MOD, while acknowledging its limitations.

**Supplementary Information:**

The online version contains supplementary material available at 10.1186/s12874-020-01205-6.

## Background

To address aggregate binary missing participant outcome data (MOD) in pairwise and network meta-analysis, the researchers usually resort to simple data-handling approaches, such as exclusion or imputation. Both approaches are popular due to their simplicity [1–3], yet notorious for the implausibility of their assumptions. A more appropriate approach, both statistically and conceptually, is to model MOD simultaneously with the observed outcomes. This approach naturally accounts for the uncertainty due to MOD, and it may also safeguard against biased results by adjusting the within-trial results (treatment effect and standard error) for MOD [4]. Since modelling of MOD does not require any data manipulation before analysis, it overrides both exclusion and imputation.

The pattern-mixture model is the most commonly described model in the methodological literature for pairwise and network meta-analysis to address binary MOD [4–7]. It consists of two parts: a model for the outcome conditional on being missing or observed and a model for the probability of MOD [8]. The pattern-mixture model incorporates an informative missingness parameter, which in the case of binary data, is known as the informative missingness odds ratio (IMOR) parameter and quantifies departures from the missing at random (MAR) assumption [4, 6, 7, 9]. The IMOR parameter is defined as the ratio between the odds of an event among MOD and the odds of an event among participants completing the trial. The IMOR parameter is naturally unknown, and we can only make clinically plausible assumptions for its value. Under the Bayesian framework, IMOR is commonly assigned a normal prior distribution in the logarithmic scale with mean and variance indicating our on average prior belief and uncertainty about the missingness mechanism, respectively [4, 6].

The pattern-mixture model can be applied under both the exact and approximate likelihood methods. The former (more frequently – but not exclusively – applied using Bayesian methods) commonly assumes within-trial binomial distribution, and thus, uses logistic regression to estimate the within-trial log ORs and their corresponding standard errors in a single step (hereafter the one-stage pattern-mixture (PM) approach). Under this approach, the log IMOR is assigned a normal prior distribution with various options regarding its structure (e.g. identical, exchangeable, or independent across trials, trial-specific, intervention-specific) rendering this approach very appealing and flexible [4, 6]. Under approximate likelihood methods (hereafter the two-stage PM approach), initially, log ORs, and standard errors are calculated in each trial after adjusting for a scenario about the missingness process (e.g. MAR as a starting point) – expressed via the mean and variance of log IMORs. Then, the adjusted log ORs are pooled using inverse-variance weighting [7, 10].

Albeit being more straightforward to apply, the two-stage PM approach has several shortcomings inherent to the within-trial normal approximation assumption. By fixing the within-trial results to the assumed mean and variance of log IMOR, the two-stage PM approach does not allow the observed data to contribute to the estimation of log IMOR to gain further insights on the missingness process in the collected trials [4]. Furthermore, the adjusted within-trial treatment effects and variances – the latter assumed known, although estimated, under the normal distribution – comprise the dataset for the second stage of the two-stage PM approach. In the presence of zero cells, continuity correction is thus required – a suboptimal approach that has been criticised for leading to biased results [11, 12]. In a typical systematic review where large and many studies are not prevalent, it is hard to justify the within-trial normal approximation [13, 14]. Consequently, the application of the two-stage PM approach may implicate the accuracy of summary results (especially, when the included trials are small, or the outcome is sparse [15]), and hence, compromise the conclusions delivered to the end-users of systematic reviews.

The advantages of the exact likelihood (one-stage approach) over the approximate normal likelihood (two-stage approach) for the synthesis of trials are well-documented in the literature for pairwise meta-analysis [16–18] and recently for network meta-analysis (NMA) [19]. However, little is known of how much the presence of MOD can challenge the behaviour of these two approaches. To our knowledge, there are only two simulation studies on the performance of the pattern-mixture approach in evidence synthesis of binary outcome data [5, 20]. However, they have considered only scenarios that allow for the approximate normality assumption. In this work, we investigate the implications of applying the one-stage and two-stage PM approaches on the relative treatment effects and the between-trial variance in NMA. In Section “Methods”, we introduce the one-stage and two-stage PM approaches for binary MOD in Bayesian random-effects NMA, and we briefly describe the empirical study. The results of the empirical study appear in the homonymous section followed by the Section “Simulation study” where we describe the set-up and the various scenarios of our simulation study. In Section “Results of the simulation study”, we present the results of the simulation study. Discussion of the findings from the empirical and simulation studies can be found in Section “Discussion”, and brief conclusions and recommendations are followed-up in Section “Conclusions.

## Methods

Consider a network of *N* trials that compare different sets of interventions regarding a binary outcome, where *a*_*i*_ represents the number of interventions (from now on called arms) investigated in trial *i* (*i* = 1, 2, …, *N*). In arm *k* = 1, 2, …, *a*_*i*_ of trial *i*, ${r}_{ik}^o$ represents the number of participants who experienced the outcome conditional on the completers (i.e. participants who completed the trial), *m*_*ik*_ represents the number of missing participants, and *n*_*ik*_ represents the total number of randomised participants.

### One-stage pattern-mixture model

By convention, ${r}_{ik}^o$ and *m*_*ik*_ are assumed to follow the corresponding binomial distributions:

$$ {r}_{ik}^o\sim Bin\left({p}_{ik}^o,{n}_{ik}-{m}_{ik}\right)\ \mathrm{and}\ {m}_{ik}\sim Bin\left({q}_{ik},{n}_{ik}\right) $$

where ${p}_{ik}^o$ is the probability of an event conditional on completers (i.e. *n*_*ik*_ − *m*_*ik*_), and *q*_*ik*_ is the probability of MOD in arm *k* of trial *i* [4, 6].

Under the pattern-mixture model, the randomised participants are distinguished to those completing and those dropping out of the trial early. Within each subgroup, the participants are further distinguished to those experiencing and those not experiencing the event. Then, the underlying probability of an event, *p*_*ik*_, can be written as a function of these subgroups using conditional probabilities [4]:

1 $$ {p}_{ik}={p}_{ik}^o\left(1-{q}_{ik}\right)+{p}_{ik}^m{q}_{ik} $$

with ${p}_{ik}^m$ being the probability of an event conditional on those dropping out of arm *k* in trial *i*. Then, the IMOR parameter is defined as follows [7]:

$$ {\delta}_{ik}=\frac{p_{ik}^m/\left(1-{p}_{ik}^m\right)}{p_{ik}^o/\left(1-{p}_{ik}^o\right)} $$

with

$$ \mathit{\log}\left({\delta}_{ik}\right)={\varphi}_{ik}\sim N\left({\omega}_{ik},{\sigma}_{ik}^2\right) $$

The relationship between ${p}_{ik}^m$ and ${p}_{ik}^o$ is explained by the formula of the IMOR parameter:

- if ${p}_{ik}^m={p}_{ik}^o$, then *δ*_*ik*_ = 1 (and *φ*_*ik*_ = 0) which suggests the MAR assumption;
- if ${p}_{ik}^m>{p}_{ik}^o$, then *δ*_*ik*_ > 1 (and *φ*_*ik*_ > 0) which suggests a deviation from the MAR assumption and indicates that the odds of an event given the missing participants are more likely than the odds of an event given the completers, and
- if ${p}_{ik}^m<{p}_{ik}^o$, then *δ*_*ik*_ < 1 (and *φ*_*ik*_ < 0) which also suggests a deviation from the MAR assumption and indicates that the odds of an event given the missing participants are less likely than the odds of an event given the completers.

In the present work, we considered independent *φ*_*ik*_ to agree with the structure of *φ*_*ik*_ in the two-stage PM approach (Section “Two-stage pattern-mixture model”):

$$ {\varphi}_{ik}\sim N\left(0,1\right) $$

where we assume on average MAR in each arm of every trial. Since the true missingness mechanism is not known, we consider the MAR assumption to be a reasonably plausible assumption following the recommendations of the relevant literature [4, 7, 9].

### Random-effects network meta-analysis model

Then, the logit function with random-effects is applied:

$$ logit\left({p}_{ik}\right)={u}_i+{\theta}_{ik} $$

$$ {\theta}_{ik}\sim N\left({\mu}_{t_{ik}{t}_{i1}},{\tau}^2\right) $$

with *u*_*i*_ = *logit*(*p*_*i*1_) being the log odds of baseline arm and *θ*_*ik*_ being the log OR of an event between arm *k* (*k* ≠ 1) and baseline arm in trial *i*. Index *t*_*ik*_ indicates the intervention studied in arm *k* of trial *i*, that is, *t*_*ik*_ ∈ {*A*, *B*, …}. Typically, *τ*^2^ is assumed common for all observed comparisons; this corresponds to a correlation equal to 0.5 between any two *θ*_*ik*_ s (with *k* ≠ 1) in a multi-arm trial [21].

Under the consistency assumption (i.e. an agreement between direct and more than one indirect source of evidence [22]), we can obtain all possible pairwise comparisons as linear combinations of the summary log ORs of the basic parameters (i.e. comparisons with the reference intervention in the network [23]):

$$ {\mu}_{jl}={\mu}_{jA}-{\mu}_{lA} $$

where A is the reference intervention in the network with a set of interventions *T* = {*A*, *B*, *C*, …} and *j* ≠ *l* ∈ *T* ∖ {*Α*} are the non-reference interventions of the network. Using the basic parameters, we can also obtain several measures of hierarchy to order the interventions from the best to worst [24]. However, intervention hierarchy is out of the scope of the present study.

### Two-stage pattern-mixture model

In the first stage, we adjust the within-trial log ORs using the pattern-mixture model (eq. (1)). In line with the one-stage PM model, we considered that log IMORs are on average MAR in each arm of every trial (i.e. *ω*_*ik*_ = 0), where *p*_*i*, *k*_ corresponds to ${r}_{ik}^o/\left({n}_{ik}-{m}_{ik}\right)$. Then, the log OR of an event between arm *k* (*k* ≠ 1) and baseline arm in trial *i* is estimated as:

$$ {y}_{ik}=\left\{\begin{array}{c} logit\left({r}_{ik}^o/\left({n}_{ik}-{m}_{ik}\right)\right)- logit\left({r}_{i1}^o/\left({n}_{i1}-{m}_{i1}\right)\right)\\ {} logit\left(\left({r}_{ik}^o+0.5\right)/\left({n}_{ik}-{m}_{ik}+1\right)\right)- logit\left(\left({r}_{i1}^o+0.5\right)/\left({n}_{i1}-{m}_{i1}+1\right)\right)\ \end{array}\right.{\displaystyle \begin{array}{c},\mathrm{no}\ \mathrm{zero}\ \mathrm{cells}\ \\ {},\mathrm{zero}\ \mathrm{cells}\ \end{array}} $$

where the term ‘zero cells’ refers to observing no events in either arm of a trial. Under the pattern-mixture model, the within-trial variance of log OR, *v*_*ik*_, is partitioned to the variance due to sampling error and to the variance arising from *φ*_*ik*_. In the present work, tο approximate the variance due to sampling error, we applied Taylor series (eq. (13) in White et al. [7]), and for the variance due to *φ*_*ik*_ we used the eq. (16) in White et al. [7] assuming zero correlation between *φ*_*ik*_ s of the compared arms in each trial and ${\sigma}_{ik}^2$ equal to 1. By convention, *v*_*ik*_ is treated as known based on the central limit theorem that trials are sufficiently large so that *y*_*ik*_ approximates the normal distribution with variance equal to *v*_*ik*_.

### Random-effects network meta-analysis model

In the second stage, following the contrast-based parameterisation described by Dias et al. [25] (Example 7(a) in the Appendix, there), in a multi-arm trial, within-trial log ORs are sampled from the following multivariate normal distribution:

$$ {\boldsymbol{y}}_i\sim {N}_{a_{i-1}}\left({\boldsymbol{\theta}}_i,{\boldsymbol{\varSigma}}_i\right) $$

with ${\boldsymbol{y}}_i={\left({y}_{i2},{y}_{i3},\dots, {y}_{i{a}_i}\right)}^{\prime }$ and ${\boldsymbol{\theta}}_i={\left({\theta}_{i2},{\theta}_{i3},\dots, {\theta}_{i{a}_i}\right)}^{\prime }$ referring to all pairwise comparisons with the baseline arm of trial *i* and

$$ {\boldsymbol{\varSigma}}_i=\left(\begin{array}{ccc}\begin{array}{c}{v}_{i2}\\ {}\mathit{\operatorname{cov}}\left({y}_{i3},{y}_{i2}\right)\end{array}& \begin{array}{c}\mathit{\operatorname{cov}}\left({y}_{i2},{y}_{i3}\right)\\ {}{v}_{i3}\end{array}& \begin{array}{c}\cdots \\ {}\cdots \end{array}\kern0.5em \begin{array}{c}\mathit{\operatorname{cov}}\left({y}_{i2},{y}_{i{a}_i}\right)\\ {}\mathit{\operatorname{cov}}\left({y}_{i3},{y}_{i{a}_i}\right)\end{array}\\ {}\vdots & \vdots & \ddots \kern0.5em \vdots \\ {}\mathit{\operatorname{cov}}\left({y}_{i{a}_i},{y}_{i2}\right)& \mathit{\operatorname{cov}}\left({y}_{i{a}_i},{y}_{i3}\right)& \begin{array}{cc}\cdots & {v}_{i{a}_i}\ \end{array}\end{array}\right) $$

being the variance-covariance matrix of trial *i* with *cov*(*y*_*ij*_, *y*_*il*_) = 1/(*n*_*i*1_*p*_*i*1_(1 − *p*_*i*1_)), *j* ≠ *l* ∈ {2, 3, …, *a*_*i*_ } which is the variance of log odds of the baseline arm (obtained using the Delta method). Then, the vector ***θ***_***i***_ of correlated random-effects in trial *i* is assumed to follow either a multivariate normal distribution (eq. (10) in Dias et al. [25]) or conditional univariate normal distributions on *θ*_*ik*_ with *k* > 2 given all other arms from 2 to *a*_*i*_ − 1 (eq. (11) in Dias et al. [25]). Using the consistency equations (Section “One-stage pattern-mixture model”), we can obtain the summary log ORs for all possible comparisons in the network.

In summary, the one-stage PM approach uses the information extracted from each arm of every trial as input data (i.e. ${r}_{ik}^o$, *m*_*ik*_, and *n*_*ik*_) and incorporates the pattern-mixture model (eq. (1)) into the hierarchical model of NMA. Contrariwise, the two-stage PM approach uses the estimated within-trial results as input data (i.e. *y*_*ik*_ and *v*_*ik*_) to perform NMA. These results have been derived by applying the pattern-mixture model (eq. (1)) under a specific assumption about *φ*_*ik*_ (‘on average MAR’ assumption, here) to obtain the *p*_*i*, *k*_.

### Factors that may affect within-trial normality approximation

We used the database of 29 networks from several health-related fields considered in previous work [6]. Detailed information on the MOD per network can be found elsewhere [6]. For this study, we considered a sample size of fewer than 50 participants to represent small trials, and event risk below 5% to be low. We characterised a network as ‘susceptible’ to within-trial normality approximation (hereinafter called ‘susceptible’ network) when there was at least one trial with a sample size less than 50 participants and/ or at least one trial-arm with observed event risk less than 5%. Otherwise, the network was characterised as ‘non-susceptible’ to within-trial normality approximation (hereinafter called ‘non-susceptible’ network). We acknowledge that these two categorisations of the networks may not be universally accepted.

### Extent and balance of MOD per trial and network

We used the ‘five-and-twenty rule’ by Sacket et al. [26], which classifies MOD in a trial as resulting in little, intermediate and serious attrition bias, alongside our definition of unbalanced MOD [6] to indicate the trial and networks as having:

- low MOD (i.e. a trial with a percentage of MOD less than 5; a network with a *median* percentage of MOD less than 5);
- moderate and balanced MOD: moderate MOD (i.e. a trial with a percentage of MOD between 5 and 20; a network with a *median* percentage of MOD between 5 and 20) which are balanced in the compared arms (i.e. a trial with a difference in the percentage of MOD in the compared arms up to 6.5; a network with a *median* difference in the percentage of MOD in the compared arms up to 6.5);
- moderate and unbalanced MOD: moderate MOD which are unbalanced in the compared arms (i.e. a trial with a difference in the percentage of MOD in the compared arms above 6.5; a network with a *median* difference in the percentage of MOD in the compared arms above 6.5);
- large and balanced MOD: large MOD (i.e. a trial with a percentage of MOD over 20; a network with a *median* percentage of MOD over 20) which are balanced in the compared arms, and,
- large and unbalanced MOD: large MOD which are unbalanced in the compared arms.

The ‘percentage of MOD for a trial’ is defined as the ratio of the number of MOD in all compared arms of the trial to the total number of randomised participants in that trial. The ‘median percentage for a network’ refers to the median of the percentage of MOD across all trials of the network.

Overall, 37% of the 539 trials in our dataset had low MOD, followed by 30% with moderate and balanced MOD, 18% with moderate and unbalanced MOD, 9% with large and unbalanced MOD, and 6% with large and balanced MOD. Almost half of the networks were classified as having moderate and balanced MOD (Table 1), followed by low MOD (41%). Overall, three networks were found to be more problematic in terms of MOD: two networks of moderate and unbalanced MOD, and one network of large and unbalanced MOD. None of the networks was classified as having large and balanced MOD.

**Table 1:** Distribution of several characteristics across networks

Characteristic	Susceptible networks^1^ (*n* = 11)	Non-susceptible networks^1^ (*n* = 18)	All networks (*n* = 29)
Total trials per network, *median (minimum, maximum)*	21 (11, 104)	9 (4, 15)	13 (4, 104)
Trials per comparison *median (minimum, maximum)*	2 (1, 13)	1 (1, 10)	1 (1, 13)
*Degree of missing outcome data (%)*			
Low *median (minimum, maximum)*	0.03 (0.00, 0.57) [2]^2^	0.02 (0.00, 0.24) [10]	0.03 (0.00, 0.57) [12]
Moderate and balanced *median (minimum, maximum)*	0.12 (0.00, 0.62) [8]	0.09 (0.00, 0.37) [6]	0.11 (0.00, 0.62) [14]
Moderate and unbalanced *median (minimum, maximum)*	0.18 (0.00, 0.45) [1]	0.09 (0.03, 0.27) [1]	0.15 (0.00, 0.45) [2]
Large and unbalanced *median (minimum, maximum)*	–	0.30 (0.03, 0.87) [1]	0.30 (0.03, 0.87) [1]
*Factors that affect within-trial normal approximation*			
Trial sample size *median (minimum, maximum)*	204 (12, 18,201)	364 (74, 8240)	262 (12, 18,201)
Event risk *median (minimum, maximum)*	0.58 (0.00, 1.00)	0.66 (0.12, 0.99)	0.60 (0.00, 1.00)
Number of zero-cells *median (minimum, maximum)*	1 (1, 4) [9]	–	1 (1, 4) [9]

^1^A network was ‘susceptible’ to within-trial normality approximation when there was at least one trial with a sample size less than 50 participants and/ or at least one trial-arm with observed event risk less than 5%

^2^Brackets indicate the number of networks with the studied characteristic

### Characteristics of the analysed networks

Eleven out of 29 networks (38%) were categorised as ‘susceptible’ and 18 as ‘non-susceptible’ (Table 1; Supplementary Table 1, Additional file 1). The former group included considerably more trials (median: 21, range: 11–104) and therefore, more trials per comparison (median: 2, range: 1–13) than the latter group (median: 9, range: 4–15 for trials; median: 1, range: 1–10 for trials per comparison) (Table 1). Of the 11 ‘susceptible’ networks, the majority (72%) had trials with moderate and balanced MOD, whereas the majority (55%) of ‘non-susceptible’ networks had trials with low MOD (Table 1). There were three networks with the most severe cases of MOD overall: one ‘susceptible’ network with moderate and unbalanced MOD, one ‘non-susceptible’ network with moderate and unbalanced MOD, and one ‘non-susceptible’ network with large and unbalanced MOD. The sample size of the trials was moderate overall (median: 204 and 364 in ‘susceptible’ and ‘non-susceptible’ networks, respectively; Table 1); however, nine of the ‘susceptible’ networks included at least one trial with less than 50 participants (Supplementary Table 1, Additional file 1). Median event risk indicated frequent events in both network categories (median: 0.58 and 0.66 in ‘susceptible’ and ‘non-susceptible’ networks, respectively; Table 1). Four of the ‘susceptible’ networks included at least one trial with an event risk of less than 5% (Supplementary Table 1, Additional file 1). Nine ‘susceptible’ networks had at least one trial with zero events or non-events (median number of zero cells: 1, range: 1–4; Table 1; Supplementary Table 1, Additional file 1).

### Model implementation and presentation of results

Both approaches were implemented in JAGS via the R-package R2jags [27] (statistical software R, version 3.6.1 [28]). Technical details on the specification of the models (i.e. prior distributions, convergence inspection, number of chains and iterations) can be found in Additional file 1. We created scatterplots to illustrate the agreement between results from the one-stage versus two-stage PM approaches for the following three model parameters: a) posterior mean of within-trial log ORs, b) posterior mean of NMA log ORs for comparisons with the reference intervention in each network, and c) the posterior median of *τ*^2^. We compared the approaches also in terms of the posterior standard deviation of the parameters mentioned above. An agreement was inferred when the points were aligned with the diagonal line. To quantify the agreement, we used the concordance correlation coefficient (CCC) [29] via the R-package epiR [30]. The R-package ggplot2 was used to draw the scatterplots [31]. The dataset and the code to perform the empirical study are available online at https://github.com/LoukiaSpin/One-stage-vs-two-stage-PM-models.git.

## Results of the empirical study

**Fig. 1:**
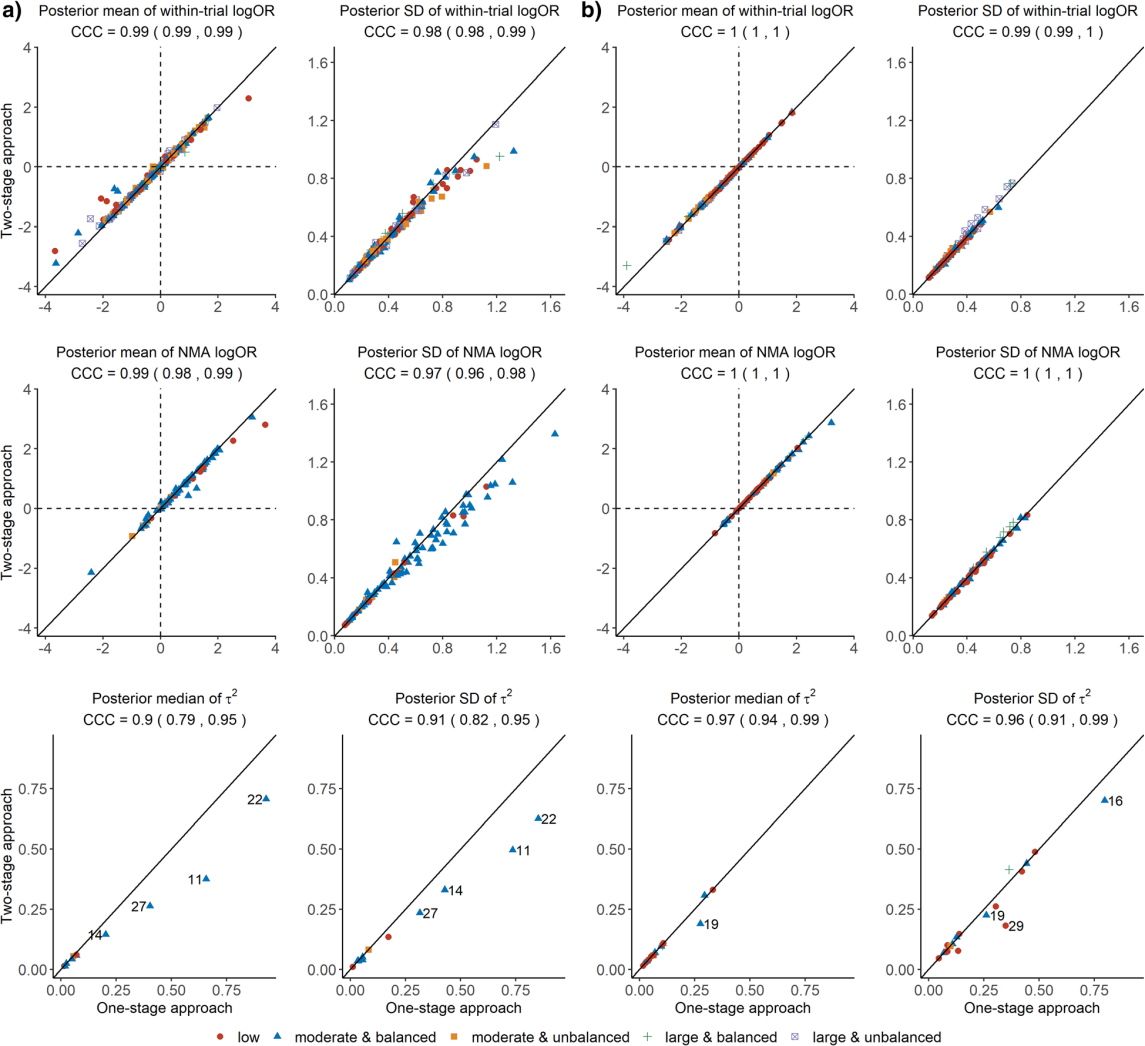
Scatterplots of the two-stage approach against the one-stage approach with regards to within-trial log OR (first row), NMA log OR (second row), and common *τ*^2^ (third row) in ‘susceptible’ networks (panel **a**)) and ‘non-susceptible’ networks (panel **b**)). Different colours indicate the degree and balance of missing outcome data across 29 networks. Results on the concordance correlation coefficient (CCC) (mean and 95% confidence interval) appear above each scatterplot. References are found in Additional file 1. NMA, network meta-analysis; log OR, odds ratio in the logarithmic scale; SD, standard deviation

The first panel of Fig. 1 shows the posterior mean and standard deviation of the within-trial log ORs across the 11 ‘susceptible’ networks (404 points, Fig. 1 a)) and 18 ‘non-susceptible’ networks (172 points, Fig. 1 b)) for a different amount of MOD. The second panel of Fig. 1 presents the posterior mean and standard deviation of the log ORs for the basic parameters of each ‘susceptible’ (104 points, Fig. 1 a)) and ‘non-susceptible’ network (80 points, Fig. 1 b)), and the third panel illustrates the posterior median and standard deviation of *τ*^2^ in the ‘susceptible’ networks (11 points, Fig. 1 a)) and ‘non-susceptible’ networks (18 points, Fig. 1 b)) for a different amount of MOD. Results on the posterior mean of residual deviance are illustrated in Additional file 1 (Table S2) to investigate whether each model fits the data satisfactorily for each network.

### Posterior mean or median

For the ‘susceptible’ networks, one-stage and two-stage PM approaches overall agreed concerning the posterior mean of within-trial log ORs (CCC: 0.99) and the posterior mean of NMA log ORs (CCC: 0.99) across the different scenarios of MOD (Fig. 1 a, first and second panel). An agreement could also be inferred for the posterior median of *τ*^2^ (CCC: 0.90), except for four networks with moderate and balanced MOD whose *τ*^2^ estimates were found to be higher under the one-stage PM approach (Fig. 1 a, third panel). In more detail, from the left to the right of the plot, the posterior median of *τ*^2^ under the two-stage PM approach was 0.14, 0.26, 0.37, and 0.71 versus 0.20, 0.40, 0.66, and 0.93 under the one-stage PM approach, respectively. These *τ*^2^ estimates corresponded to moderate statistical heterogeneity (network 14; the posterior median of *τ*^2^ was lower than the third quartile of the corresponding predictive distribution for *τ*^2^) and large statistical heterogeneity (networks 11, 22, and 27; the posterior median of *τ*^2^ was larger than the third quartile of the corresponding predictive distributions for *τ*^2^). Note that the remaining ‘susceptible’ networks had low statistical heterogeneity as the posterior median of *τ*^2^ was lower than the median of the corresponding predictive distributions for *τ*^2^. Therefore, in ‘susceptible’ networks with large statistical heterogeneity, the compared approaches did not agree in the estimation of *τ*^2^ as the estimated *τ*^2^ tended to be larger under the one-stage PM approach when compared with the two-stage PM approach.

Contrariwise, in ‘non-susceptible’ networks, the compared approaches perfectly agreed concerning the posterior mean of within-trial log ORs and the posterior mean of NMA log ORs across the different scenarios of MOD (Fig. 1 b, first and second panel). The agreement was almost perfect for the posterior median of *τ*^2^ (CCC: 0.97) apart from one network with moderate and balanced MOD that showed a slightly larger posterior median of *τ*^2^ under the one-stage PM approach (Fig. 1 b, third panel). Specifically, the *τ*^2^ estimates were 0.19 and 0.27 under the two-stage PM approach and one-stage PM approach, respectively – both estimates indicated moderate statistical heterogeneity for being lower than the third quartile of the selected predictive distribution for *τ*^2^.

### Posterior standard deviation

In ‘susceptible’ networks, the posterior standard deviation of NMA log ORs was systematically larger under the one-stage PM approach, especially in networks with moderate and balanced MOD (Fig. 1 a, second panel). This was expected as the one-stage PM approach accounted for the uncertainty in the estimation of all parameters in the pattern-mixture model (eq. (1)). Therefore, the uncertainty increased when the available information was limited; namely, the included trials were small with low events and substantial MOD. Contrariwise, in ‘non-susceptible’ networks, the agreement was almost perfect for the posterior standard deviation of NMA log ORs (Fig. 1 B, second panel).

Regarding the posterior standard deviation of *τ*^2^, the agreement was higher in ‘non-susceptible’ networks overall (CCC: 0.96, 95% confidence interval (CI): 0.91 to 0.99) as compared with the ‘susceptible’ networks (CCC: 0.91, 95% CI: 0.82 to 0.95). In the ‘susceptible’ networks, the one-stage PM approach resulted in a larger posterior standard deviation of *τ*^2^ for the four networks mentioned above (Section “Posterior mean or median”) (Fig. 1 a and b, third panel). Therefore, in ‘susceptible’ networks with large statistical heterogeneity, the one-stage PM approach tended to estimate *τ*^2^ also with larger uncertainty as compared to the two-stage PM approach.

## Simulation study

We simulated 1000 triangle networks of two-arm trials and three interventions: new intervention, old intervention, and placebo. Our main interest was the comparison of the former two interventions; however, for completeness, we also presented the results on the basic parameters (i.e. comparisons with placebo). The ultimate goal of the simulation study was to compare the performance of the two PM approaches under a setting where the normality approximation is compromised (i.e. small trials with low events) while considering the recommended ‘on average MAR’ assumption as a primary analysis to model informative MOD. In practice, it is more plausible for MOD to be informative; however, note that we cannot know the exact missingness mechanism.

### Simulation set-up

The simulation set-up was in line with a previous study on MOD in NMA [5]. Briefly, we assumed a larger *beneficial* underlying log OR for ‘new intervention versus placebo’ as compared to ‘old intervention versus placebo’ and we used the consistency equation to obtain the underlying log OR for ‘new versus old intervention’ (Table 2). To generate the number of events in each arm of every trial, we considered the data-generating model of Hartung and Knapp for a random-effects pairwise meta-analysis [34]. For a brief description of the data-generating model, the reader can refer to Additional file 1. To obtain the event risks among the completers in each arm of every trial, we used the linkage function of Turner et al. [4] (eq. (7), there) that is a function of the IMOR parameter, the underlying event risks and the probability of MOD in each arm of every trial.

**Table 2:** Scenarios for the simulation set-up

*Number of trials per comparison*	
typical loop	*NO* = 1, *NP* = 3, *OP* = 4
*Trial size (*${n}_{i,k}^E={n}_{i,k}^C={n}_i$ *in trial i)*	
< 50 (small)	*n*_*i*_~*U*(12, 39) placebo-controlled trials
	*n*_*i*_~*U*(15, 49) old-controlled trials
> 100 (moderate)	*n*_*i*_~*U*(102, 187) placebo-controlled trials *n*_*i*_~*U*(128, 241) old-controlled trials
*Initial event rates of the control arm in trial i*	
low events	${p}_{i,P}^{C,0}\sim U\left(0.05,0.09\right)$ placebo-controlled trials
	${p}_{i,O}^{C,0}\sim U\left(0.10,0.15\right)$ old-controlled trials
frequent events	${p}_{i,P}^{C,0}\sim U\left(0.27,0.40\right)$ placebo-controlled trials
	${p}_{i,O}^{C,0}\sim U\left(0.63,0.76\right)$ old-controlled trials
*Unbalanced risk of missing outcome data (*${q}_{i,k}^E<{q}_{i,k}^C$ *in trial i)*	
moderate	${q}_{i,k}^E\sim U\left(0.05,0.10\right)$, ${q}_{i,k}^C\sim U\left(0.11,0.20\right)$
large	${q}_{i,k}^E\sim U\left(0.21,0.30\right)$, ${q}_{i,k}^C\sim U\left(0.31,0.40\right)$
*Missingness mechanisms* via *log IMOR*	
informative	*φ*_*i*, *P*_~*TN*(*μ* = − *log* (2), *σ*^2^ = 1, *a* = *log* (1))
	*φ*_*i*, *k*_~*TN*(*μ* = *log* (2), *σ*^2^ = 1, *a* = *log* (1)) *k* = *N*, *O*
*Treatment effects*	
basic parameters	*LOR*_*NP*_ = *ln* (2), *LOR*_*OP*_ = *ln* (1.5)
functional parameter	*LOR*_*NO*_ = *LOR*_*NP*_ − *LOR*_*OP*_ (consistency equation)
*Common between-trial variance*	
predictive distribution	*τ*^2^~*LΝ*(−3.95, 1.34^2^) (small) *τ*^2^~*LΝ*(−2.56, 1.74^2^) (substantial)

Note: *C* Control; *E* Experimental arm; *IMOR* Informative missingness odds ratio; *LN* Log-normal distribution; *LOR* Log odds ratio; *N* New intervention; *O* Old intervention; *P* Placebo; *T* Truncated normal distribution; *U* Uniform distribution

Typical loop as defined by Veroniki et al. [32]

Using predictive log-normal distributions that correspond to all-cause mortality and generic health setting for small and substantial between-trial variance, respectively [33]

### Simulation scenarios

In the present work, we considered only a ‘typical loop’ with one trial comparing ‘new versus old intervention’, three trials comparing a ‘new intervention with placebo’, and four trials comparing an ‘old intervention with placebo’ [32] (Table 2). The simulation scenarios were constructed such that to explore the impact of four key factors: the sample size of the trials, frequency of events, the extent of MOD, and degree of *τ*^2^. With respect to sample size, we considered a trial as having a small sample size if *n* < 50, and moderate sample size if *n* > 100, equally distributed in the compared arms (Table 2). For event risk at the control arm, a maximum of 15% was considered to be low, and at least 27% was considered to be frequent (Table 2). Initially, we considered a maximum of 5% as low event risk at the control arm. However, this scenario resulted in generating networks with zero events in both arms for the majority of trials, particularly for the scenario of fewer than 50 participants, and thus, creating serious convergence issues in both approaches. We focused on scenarios of unbalanced MOD with more MOD in the control arm and cases of moderate and large MOD (Table 2). A previous study revealed that moderate and large MOD (which were unbalanced in the compared arms) affected the performance of the one-stage PM approach in terms of the posterior standard deviation of log OR and *τ*^2^ [5]. We considered informative missingness process in all interventions: IMOR equal to 2 for the new and old interventions (i.e. the odds of an event given MOD are twice the odds of an event given the completers) and IMOR equal to 1/2 for placebo. We considered *τ*^2^ equal to 0.02 and 0.07 to reflect small and substantial true statistical heterogeneity, respectively. These values correspond to the median of the predictive log-normal distribution for all-cause mortality (95% prior interval: 0.001–0.26) and generic health setting (95% prior interval: 0.002–2.67), respectively [33]. Table 2 illustrates the scenarios considered in the simulation.

### Model implementation and presentation of results

For each of the 16 scenarios (4 factors of two categories), we performed a Bayesian random-effects NMA with consistency equations using the one-stage and two-stage PM approaches to analyse the generated networks. All analyses were performed under the ‘on average MAR’ assumption as the recommended primary analysis [4, 7, 9, 35, 36]. In line with the empirical study, we considered a non-informative normal prior distribution with zero mean and variance equal to 10,000 on all location parameters for both PM approaches. We assigned a predictive prior distribution on *τ*^2^ that refers to the improvement of symptoms for pharmacological versus placebo comparison (median: 0.11, 95% prior interval: 0.01–2.13) and aligns with the beneficial outcome considered in the simulation study [33]. We preferred this prior distribution to a weakly-informative prior distribution, such as half-normal prior distribution on *τ* with variance one (median: 0.67, 95% prior interval: 0.03–2.24), as the latter compromised the estimation of the parameters for the scenario of low events (Supplementary Table 3–6, Additional file 1).

For each scenario, we calculated the bias for (NMA) log OR as the difference between the posterior mean of log OR and the underlying log OR. The bias for *τ*^2^ was calculated as the difference between the posterior median of *τ*^2^ and the underlying *τ*^2^. The posterior width of 95% credible interval (CrI) for a parameter (log OR or *τ*^2^) was calculated as the difference between the 97.5 and 2.5% percentile of the simulated parameter. The bias of the posterior mean and the width of the 95% CrIs of log OR for every comparison are illustrated using dot plots. The posterior mean and posterior standard deviation of log ORs are presented in tables in the Additional file 1 (Supplementary Table 7–9). The posterior median and posterior standard deviation of *τ*^2^ alongside the bias and the width of the 95% CrI are presented in Table 3. Regarding the bias and the width of the 95% CrIs of log OR, we presented only the results for small *τ*^2^ as the behaviour of the compared approaches was similar under small and substantial true *τ*^2^.

**Table 3:** Posterior median (and 95% CrI) and bias (and width of 95% CrI) for common *τ*^2^

small *τ*^2^		moderate MOD		large MOD	
trial size	frequency	one-stage	two-stage	one-stage	two-stage
small	low	0.13 (6 × 10^− 3^, 2.59) 0.11 (2.58)	0.10 (6 × 10^− 3^, 1.14) 0.08 (1.13)	0.13 (6 × 10^− 3^, 3.06) 0.11 (3.06)	0.10 (6 × 10^− 3^, 1.20) 0.08 (1.19)
moderate	low	0.07 (4 × 10^− 3^, 0.70) 0.05 (0.70)	0.07 (5 × 10^− 3^, 0.64) 0.05 (0.63)	0.08 (5 × 10^− 3^, 0.97) 0.07 (0.96)	0.08 (5 × 10^− 3^, 0.82) 0.06 (0.81)
small	frequent	0.09 (5 × 10^− 3^, 0.94) 0.07 (0.93)	0.08 (5 × 10^− 3^, 0.83) 0.06 (0.82)	0.09 (5 × 10^− 3^, 1.04) 0.07 (1.03)	0.09 (6 × 10^− 3^, 0.91) 0.06 (0.90)
moderate	frequent	0.04 (3 × 10^− 3^, 0.31) 0.02 (0.31)	0.04 (4 × 10^− 3^, 0.32) 0.02 (0.31)	0.05 (4 × 10^− 3^, 0.40) 0.03 (0.40)	0.05 (4 × 10^− 3^, 0.42) 0.03 (0.42)
substantial *τ*^2^		moderate MOD		large MOD	
trial size	frequency	one-stage	two-stage	one-stage	two-stage
small	low	0.13 (6 × 10^− 3^, 2.52) 0.05 (2.51)	0.10 (6 × 10^− 3^, 1.16) 0.02 (1.15)	0.14 (6 × 10^− 3^, 3.29) 0.06 (3.29)	0.10 (6 × 10^− 3^, 1.21) 0.02 (1.21)
moderate	low	0.09 (5 × 10^− 3^, 1.05) 0.01 (1.05)	0.08 (5 × 10^− 3^, 0.86) 0.004 (0.85)	0.09 (5 × 10^− 3^, 1.17) 0.02 (1.17)	0.08 (5 × 10^− 3^, 0.90) 0.01 (0.89)
small	frequent	0.10 (5 × 10^− 3^, 1.15) 0.02 (1.14)	0.09 (6 × 10^− 3^, 0.95) 0.01 (0.95)	0.10 (5 × 10^− 3^, 1.26) 0.02 (1.25)	0.09 (6 × 10^− 3^, 1.01) 0.01 (1.01)
moderate	frequent	0.06 (4 × 10^− 3^, 0.57) −0.02 (0.57)	0.05 (4 × 10^− 3^, 0.56) − 0.02 (0.56)	0.06 (4 × 10^− 3^, 0.58) − 0.02 (0.57)	0.06 (4 × 10^− 3^, 0.56) − 0.02 (0.56)

*MOD* Missing outcome data

Posterior median and 95% CrI (in parenthesis) are provided in the first line of every cell, followed by bias and width of 95% CrI (in parenthesis) in the second line of every cell

To demonstrate that there is an association between the within-trial log OR and its standard error, when normality approximation cannot be defended (i.e. small trials with low events), we used the simulated triangles to estimate the covariance between the within-trial log OR and its standard error at the first stage of the two-stage PM approach. We created a scatterplot for each scenario where we plotted the estimated within-trial standard error of log OR against the within-trial log OR, and we used different colours to illustrate the magnitude of covariance. We presented the results for ‘new versus old intervention’ in the main text and the results for the comparisons with placebo as Supplementary Figs. 1–2 (Additional file 1).

For each simulation, we used three parallel chains with different initial values; thinning equal to 10; 80,000 updates; and a burn-in of 20,000 Markov chain Monte Carlo samples. Simulations and analyses were performed in R [28]. The dot plots and scatterplots were created using the R-package ggplot2 [31]. The code and necessary material to generate and analyse the triangles are available online at https://github.com/LoukiaSpin/One-stage-vs-two-stage-PM-models.git.

## Results of the simulation study

### Bias and width of 95% credible interval of log ORs

**Fig. 2:**
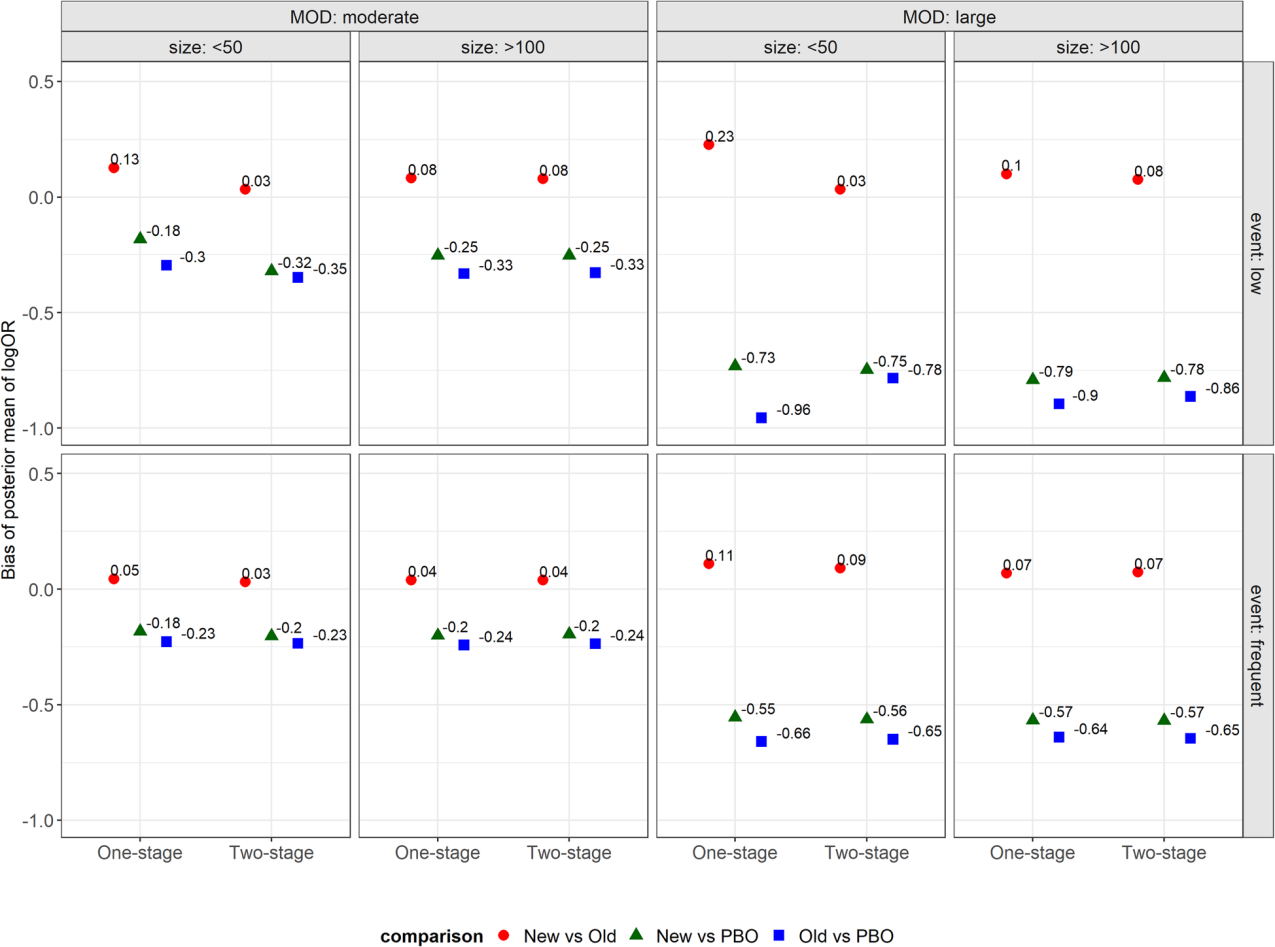
Dot plots on the bias of posterior mean of NMA log OR for all pairwise comparisons under one-stage and two-stage approaches while accounting for the degree of missing outcome data (moderate, large) being unbalanced in the compared arms, the size of trials (small, moderate), the event frequency (low, frequent) and small *τ*^2^. MOD, missing outcome data

For the case of a low event and small trial size, we encountered small-scale convergence issues for the log OR of all comparisons under the one-stage PM approach alone (1 to 4% of simulations whose results were discarded). In both approaches, the absolute bias of the posterior mean of log OR for ‘new versus old intervention’ was smaller under all scenarios as compared to the bias of posterior mean of log OR for both basic parameters (Fig. 2). The one-stage PM approach overestimated the posterior mean of log OR for ‘new versus old intervention’ in the presence of small trials with low event frequency, and notably, for large MOD (Fig. 2). On the contrary, the bias in the two-stage PM approach was very low for those scenarios (bias equal to 0.03). In the remaining scenarios, the bias of the posterior mean of log OR for ‘new versus old intervention’ was similar in both approaches.

Interestingly, the posterior mean of the log OR for both basic parameters was substantially underestimated in both approaches in the presence of large MOD (Fig. 2). For a low event and small trial size, both basic parameters had a smaller bias under the one-stage PM approach. The exception was the case of large MOD, where the log OR for ‘old intervention versus placebo’ was slightly more biased under the one-stage approach (Fig. 2; Supplementary Table 8–9, Additional file 1). In the remaining scenarios, the bias of the posterior mean of log OR for the basic parameters was similar in both models (Fig. 2; Supplementary Table 8–9, Additional file 1).

The relatively high negative bias in the basic parameters under both approaches may be attributed to the residual bias after considering the MAR assumption to analyse informative MOD, which were assumed to be moderate or large in all included trials. To investigate whether the extent of MOD may indeed explain this extent of bias, we re-ran the simulation study also considering low attrition bias (%MOD < 5) in all included trials. Under this best-case situation, the bias in log OR of the basic parameters was reduced in both approaches. Specifically, the bias ranged from − 0.1 (moderate trial size with frequent events and substantial *τ*^2^) to 0.07 (small trials with a low event and small *τ*^2^) under the one-stage PM approach, and from − 0.19 (small trials with a low event and substantial *τ*^2^) to − 0.05 (frequent events and small *τ*^2^) under the two-stage PM approach (Supplementary Fig. 3, Additional file 1). Therefore, increasing the amount of MOD increased the bias in both basic parameters, particularly under the two-stage PM approach. Note that in each network of our database, the percentage of MOD (%MOD) was ranging from very low levels (indicating low attrition bias; %MOD < 5) to moderate or large levels (indicating serious attrition bias; %MOD > 20) across the trials. Thus, we consider our simulation study to reflect a rather worst-case situation; in a ‘typical’ network, the bias in the log OR of the basic parameters would be at lower levels.

**Fig. 3:**
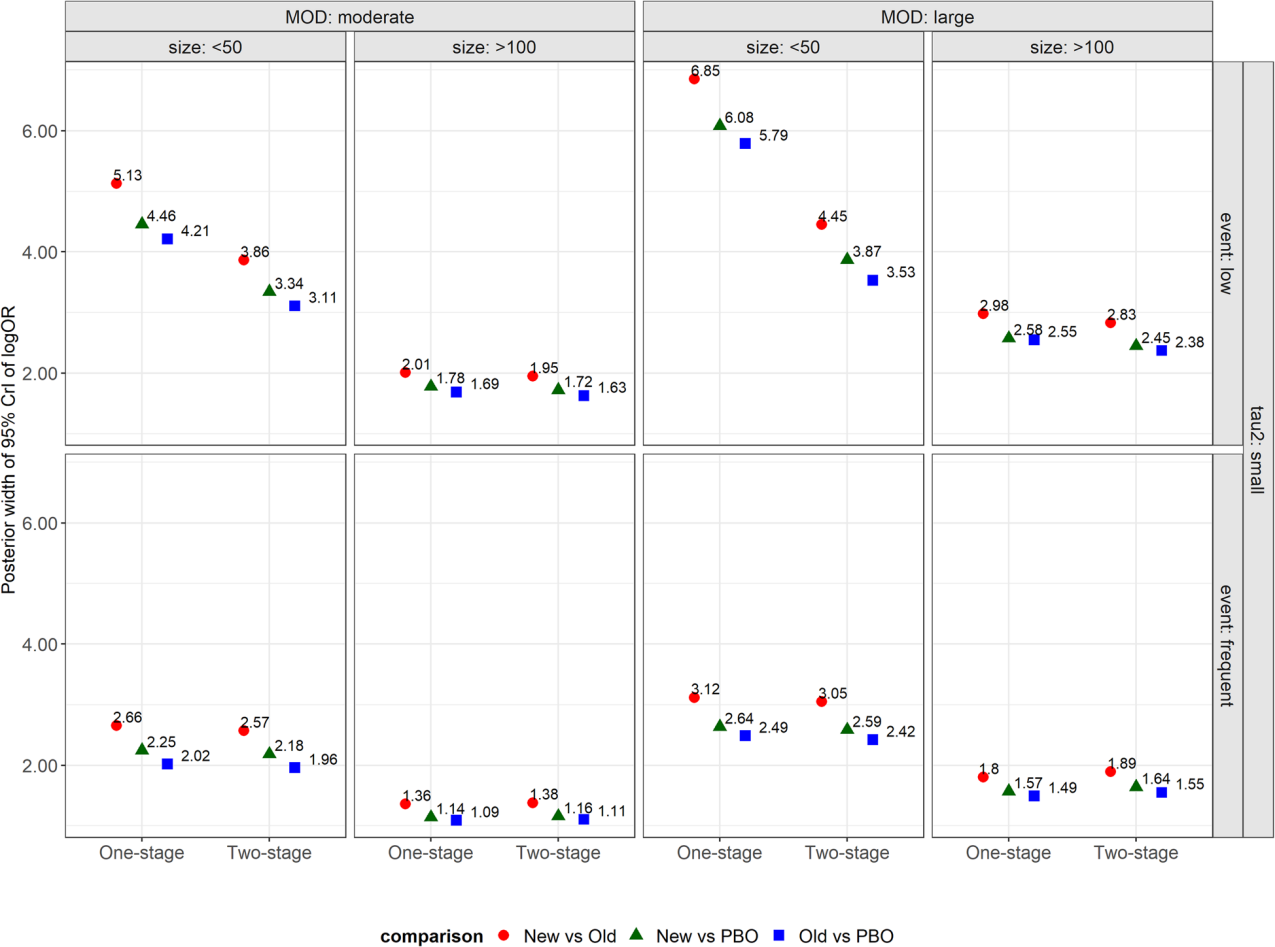
Dot plots on the width of 95% credible interval of NMA log OR for all pairwise comparisons under one-stage and two-stage approaches while accounting for the degree of missing outcome data (moderate, large) being unbalanced in the compared arms, the size of trials (small, moderate), the event frequency (low, frequent) and small *τ*^2^. MOD, missing outcome data

The 95% CrIs of log OR were wider in the one-stage PM approach for all comparisons – especially, for small trials with low events, and large MOD (range: 5.79–6.85 under the one-stage PM approach; range: 3.53–4.45 under the two-stage PM approach) (Fig. 3). Under these scenarios, the available information was limited, and therefore, both approaches estimated log OR with greater uncertainty as compared to scenarios with more information (e.g. moderate trial size and/ or frequent events). However, since the one-stage PM approach inherently treats all parameters of the pattern-mixture model (eq. (1)) as random variables, the uncertainty around the estimation of log OR was larger under this approach in ‘susceptible’ networks with considerable MOD. Contrariwise, the two-stage PM approach estimated the within-trial log ORs and their standard error at the first stage (via the pattern-mixture model). Therefore, the two-stage PM approach ‘disregarded’ the uncertainty in the estimation of the within-trial log ORs at the second stage leading to spuriously more precise summary log ORs even in the presence of large MOD.

### Ad hoc analysis: association between the within-trial log OR and its standard error

**Fig. 4:**
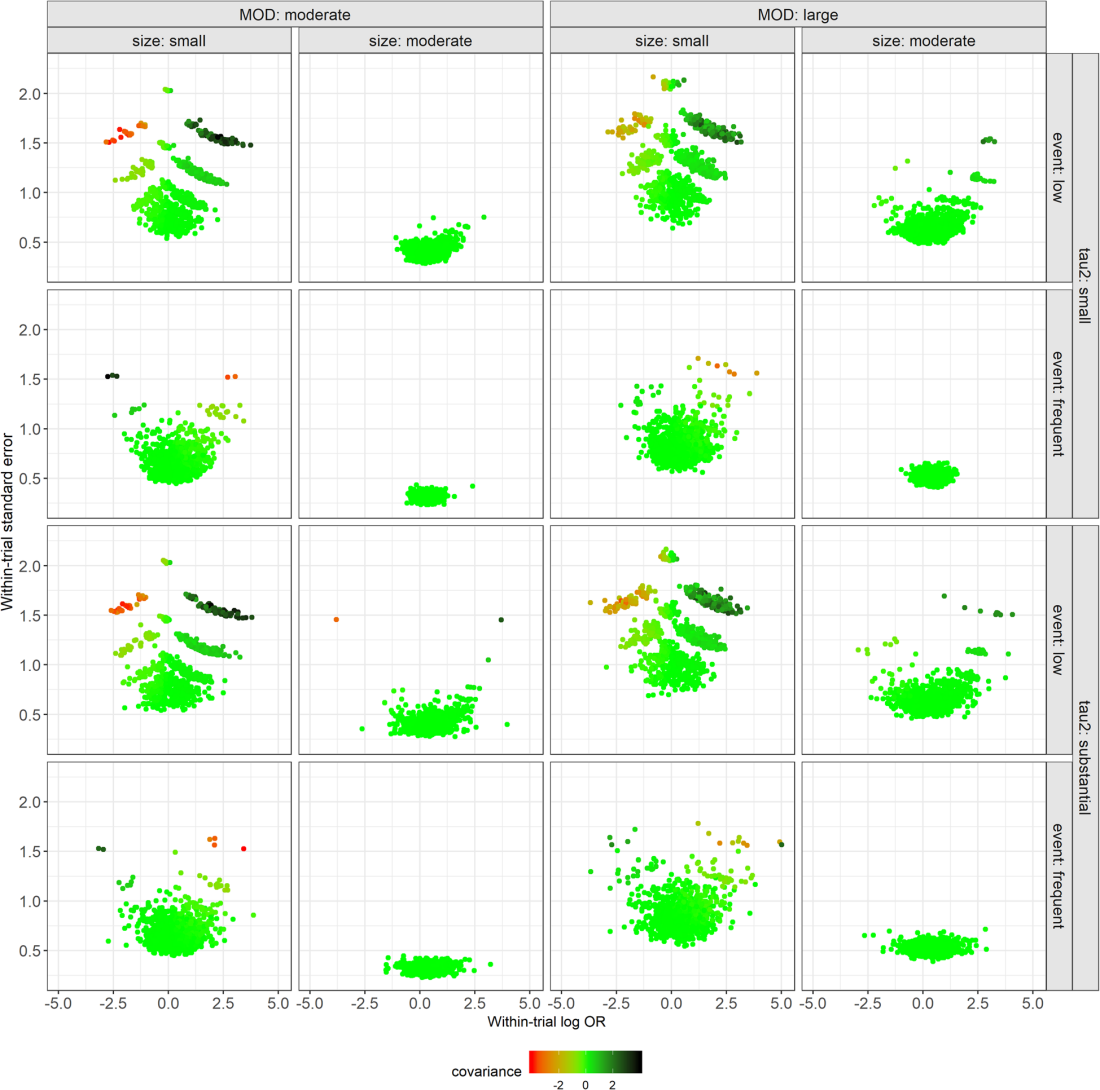
A panel of scatterplots on the within-trial standard error of log OR for ‘new versus old intervention’ (axis *y*) against the within-trial log OR for that comparison (axis *x*) for each simulation scenario. The colour key indicates the magnitude of covariance between the within-trial standard error of log OR and within-trial log OR for that comparison. MOD, missing outcome data; OR, odds ratio

Figure 4 illustrates a panel of scatterplots on the within-trial standard error of log OR for ‘new versus old intervention’ (*y*) against the within-trial log OR for that comparison (*x*) for each simulation scenario. For positive values of *x*, the covariance between *x* and *y* was positive, and therefore, trials with larger positive *x* corresponded to larger *y* and received smaller weight, whereas trials with smaller positive *x* corresponded to smaller *y* and received larger weight. On the contrary, for negative values of *x*, the covariance between *x* and *y* was negative, and therefore, bias was upwards for the pooled log OR. This pattern was observed for trials with small size and/ or low event frequency, regardless of *τ*^2^, and became more evident for large MOD (Fig. 4). The conclusions were the same for the comparison of new and old intervention versus placebo (Supplementary Fig. 1–2, Additional file 1).

### Bias and width of 95% credible interval of common τ^2^

Both approaches achieved convergence in all scenarios regarding *τ*^2^. Under small true *τ*^2^, both approaches estimated a similarly low posterior median of *τ*^2^ for moderate trial size and frequent events that approached the truth regardless of the MOD scenario (Table 3). In the remaining scenarios, both approaches overestimated *τ*^2^ similarly for moderate and large MOD, though the bias was slightly larger under the one-stage PM approach (from 0.05 to 0.11) as compared to the two-stage PM approach (from 0.05 to 0.08), especially, for small trials with a low event. The overestimation may be attributed to having a small true *τ*^2^ which has the same likelihood as the first quartile of the prior predictive distribution that we assigned on *τ*^2^ in both approaches (equal to 0.02), and thus, *τ*^2^ was overestimated.

The conclusions were similar for substantial true *τ*^2^ (Table 3). As expected, the posterior median of *τ*^2^ was slightly larger in both approaches in most scenarios compared to the posterior median of *τ*^2^ under small true *τ*^2^. However, bias was lower under substantial true *τ*^2^ in all scenarios as compared to small true *τ*^2^. A plausible explanation may be that the substantial true *τ*^2^ was closer to the median of the prior predictive distribution for *τ*^2^ in both approaches (equal to 0.11), and hence, the magnitude of overestimation was relatively smaller under substantial true *τ*^2^ than under small true *τ*^2^.

Overall, the one-stage PM approach led to wider 95% CrIs for *τ*^2^ as compared to the two-stage PM approach, especially for small trials with low events (Table 3). As expected, in both approaches, 95% CrIs for *τ*^2^ were wider under substantial *τ*^2^ (range: 0.57–3.29 in the one-stage PM approach; 0.56–1.21 in the two-stage PM approach) when compared with small *τ*^2^ (range: 0.31–3.06 in the one-stage PM approach; 0.31–1.19 in the two-stage PM approach) as well as under large MOD (range: 0.40–3.29 in the one-stage PM approach 0.42–1.21 in the two-stage PM approach) as compared to moderate MOD (range: 0.31–2.58 in the one-stage PM approach; 0.31–1.15 in the two-stage PM approach). In the case of moderate trial size with frequent events, both approaches led to a very similar width of 95% CrI for *τ*^2^ regardless of the MOD scenario.

## Discussion

We compared the one-stage approach with the two-stage approach in the presence of MOD via the pattern-mixture model using Bayesian random-effects NMA. We performed an empirical and simulation study to investigate the behaviour of NMA log OR and *τ*^2^ under moderate or large MOD and design-factors that implicate the within-trial approximate normality assumption in the two-stage approach (i.e. sample size and event frequency).

The empirical study revealed that in the case of ‘susceptible’ networks with moderate MOD, the posterior standard deviation of NMA log OR was systematically larger under the one-stage PM approach. The simulation study indicated that this behaviour was more evident in the presence of small trials with low events and exacerbated for large MOD. This is a situation where the available information is limited, and therefore, the uncertainty around the estimated NMA log OR increases. Our results are in line with Stijnen et al. [17] – albeit the authors applied binomial-normal and hypergeometric-normal models in the absence of MOD.

Furthermore, the empirical study did not indicate any systematic differences in the posterior mean of within-trial log ORs and NMA log ORs (for the basic parameters) between the compared approaches across the different amounts of MOD. Nevertheless, the simulation study revealed that in networks of small trials with low events and large MOD, the one-stage PM approach resulted in a relatively higher positive bias of NMA log OR for ‘new versus old intervention’ (functional parameter) as compared to the two-stage PM approach. This behaviour may be an artefact of the consistency equation, which is implicit also for the bias of NMA log OR for ‘new versus old intervention’ (see, Additional file 1). Presenting only the simulation results for the functional parameters of interest may be misleading if there is substantial bias in at least one of the basic parameters. This is because the bias on the functional parameters may be cancelled out to a great extent through the consistency equation, especially, in the two-stage PM approach.

In the presence of large statistical heterogeneity, the empirical study revealed that the one-stage PM approach tended to provide a larger estimate of *τ*^2^ as compared to the two-stage PM approach for ‘susceptible’ networks. This observation also concurred with moderate and balanced MOD. A previous study [5] and the present simulation study did not indicate any implications of the amount of MOD on the estimation of *τ*^2^; though, large MOD led to greater uncertainty in the estimation of *τ*^2^ – we observed this behaviour in our simulation study – more notably for the one-stage PM approach [5]. However, our simulation study did not reveal the same large discrepancy in the compared approaches concerning the estimation of *τ*^2^ in networks of small trials with a low event and substantial true *τ*^2^. A plausible explanation may be that the substantial true *τ*^2^ was much lower than the estimated *τ*^2^ in the empirical study (minimum equal to 0.20) to be able to capture a larger discrepancy in the compared approaches. Both true values for *τ*^2^ referred to a ‘typical’ meta-analysis with small or substantial statistical heterogeneity. Therefore, we consider the four networks with a large estimation of *τ*^2^ under the one-stage PM approach to representing a rather extreme situation.

The parameter *τ*^2^ is a nuisance parameter in the random-effects model, and it has no intuitive clinical interpretation as opposed to log OR. Nevertheless, *τ*^2^ is an important parameter for the evaluation of the certainty of the evidence in the context of inconsistency using the GRADE framework [37, 38]. The magnitude of *τ*^2^ affects our decision to downgrade (and by how many levels) or not the evidence for inconsistency: the larger the *τ*^2^ the more likely to downgrade the evidence for the investigated outcome. Therefore, how much accurately *τ*^2^ is estimated in a model is of critical importance. Our simulation study revealed that both approaches had a similar behaviour overall, except for networks of small trials with a low event where the one-stage PM approach led to a slightly larger bias in the estimation of *τ*^2^. Nonetheless, this should not be viewed as a reason to prefer the two-stage PM approach over the one-stage PM approach, because, in such networks, the two-stage PM approach cannot be reliable for relying on the normality approximation.

The ignorance of the inherent correlation between the within-trial log OR and within-trial standard error in conjunction with considerable MOD also raises concerns for the credibility of the results from the two-stage PM approach, particularly, in networks of small trials with low event frequency [15, 39]. As already illustrated in Fig. 4, there is a positive association between the within-trial log OR and its standard error when the within-trial log ORs are positive. However, there is a negative association when the within-trial log ORs are negative, and this pattern was obvious in networks of small trials with a low event. Stijnen et al. [17] noted that a positive or negative association between within-trial log OR and its standard error would result in a downward or upward bias in log OR, respectively. In our study, this implication was obvious only for the basic parameters. As we already mentioned, implying consistency in the bias for the ‘new versus old intervention’ led to smaller (yet positive) bias when compared with the bias for the basic parameters.

The flexibility of the one-stage PM approach comes at a high computational cost as it appeared 10-fold more computationally exhaustive compared with the two-stage PM approach. Not surprisingly, convergence issues occurred for the estimation of the NMA log OR in the networks of small trials and low event frequency only under the one-stage PM approach. The use of continuity correction seems to aid the convergence of the two-stage PM approach for the NMA log OR in this particular scenario. Nevertheless, both approaches share a common limitation: the assumption of normally distributed random-effects which, if deemed inappropriate (e.g. there are outlying trials in the synthesised dataset [15]), may compromise the validity of the results [17]. Using a simulation study to compare seven models for random-effects meta-analysis in the frequentist framework, Jackson et al. [18] demonstrated that both the binomial-normal (one-stage approach) and normal-normal (two-stage approach) models performed poorly overall. The authors suggested alternative model parameterisations (models 4, 6 and 7, there) especially when the event is low or there is considerable statistical heterogeneity according to visual inspection of the forest plot. Extending these models to incorporate the pattern-mixture model in the Bayesian framework may be proper alternatives to the current one-stage and two-stage PM approaches.

We did not perform sensitivity analysis to different assumptions about the missingness mechanisms in the compared interventions, as we were interested in investigating the performance of the competing models in the presence of MOD, rather than inferring on the relative effectiveness of the compared interventions in the studied networks. We have investigated the performance of the competing models assuming MAR, which is the recommended starting point according to the relevant published literature [4, 7, 9, 35, 36]. The competing models will have the same behaviour regarding their performance under the same assumption of informative missingness. To raise awareness for good practice in the analysis of MOD, we advise the researchers to systematically apply a sensitivity analysis to a series of gradually stringent yet clinically plausible scenarios for the missingness mechanisms in the compared interventions to investigate whether inferences deviate from the MAR assumption, the recommended primary analysis.

Furthermore, in the empirical study, we have not performed a sensitivity analysis to different prior distributions for the between-trial variance, as we are not interested in the relative effectiveness of the competing interventions in the analysed networks but in the performance of the competing models. Our simulation study revealed that the competing models maintained their performance under the weakly-informative prior (Tables S3-S5 in Additional file 1). However, the posterior standard deviation increased in both models, when compared with the results under the predictive prior (Tables S7-S9 in Additional file 1), particularly for scenarios that compromised the approximate normality assumption, as expected. In the Bayesian analysis, it is a good practice to consider different plausible prior distributions for the between-trial variance that align with the type and frequency of the investigated outcome as well as the intervention-comparison type. Then, the researchers can investigate the sensitivity of conclusions from the primary analysis to different prior distributions for the between-trial variance. The recently updated NICE Guide to the Methods of Technology Appraisal provides recommendations for selecting the appropriate prior distribution for the between-trial variance in NMA [40].

## Conclusions

The two-stage PM approach is straightforward to implement for having easier parameterisation, no convergence issues, and shorter convergence time as compared to the one-stage PM approach. Nevertheless, the well-known statistical shortcomings of this approach that relate to its approximate normal likelihood assumption and the inability to learn about the missingness mechanisms (since the missingness parameter is fixed rather than estimated) render this approach less appealing overall for the analysis of MOD. The one-stage PM approach tackles these limitations, and thus, it may be considered as a more appropriate approach. However, the simulation study failed to demonstrate the one-stage PM approach as superior due to considerable bias in the NMA log ORs, especially for large MOD, which can be slightly lower or similar to the corresponding bias under the competing approach. Until a more competent model is developed, we advise the researchers to apply the one-stage PM approach to handle MOD, especially in situations that make the approximate normality assumption difficult to defend, provided that the limitations of this approach (as demystified in the present empirical and simulation study) are fully acknowledged in the discussion of the NMA results.

## Supplementary Information

**Additional file 1: Table S1**. Comparison size and factors that affect within-trial normal approximation. **Table S2**. The posterior mean of residual deviance of each model per network. **Table S3**. Posterior mean (95% CrI) and bias (width of 95% CrI) for log OR (new versus old) under half-normal prior distribution on τ. **Table S4**. Posterior mean (95% CrI) and bias (width of 95% CrI) for log OR (new versus placebo) under half-normal prior distribution on τ. **Table S5**. Posterior mean (95% CrI) and bias (width of 95% CrI) for log OR (old versus placebo) under half-normal prior distribution on τ. **Table S6**. Posterior median (95% CrI) and bias (width of 95% CrI) for common ***τ***^**2**^ under half-normal prior distribution on τ. **Table S7**. Posterior mean (95% CrI) and bias (width of 95% CrI) for log OR (new versus old intervention) under empirical prior distribution on τ. **Table S8**. Posterior mean (95% CrI) and bias (width of 95% CrI) for log OR (new vs placebo) under empirical prior distribution on τ. **Table S9**. Posterior mean (95% CrI) and bias (width of 95% CrI) for log OR (old versus placebo) under empirical prior distribution on τ. **Fig. S1**. A panel of scatterplots on the within-trial standard error of log OR for ‘new intervention versus placebo’ (axis *y*) against the within-trial log OR for that comparison (axis *x*) for each simulation scenario. The colour key indicates the magnitude of covariance between the within-trial standard error of log OR and within-trial log OR for that comparison. MOD, missing outcome data; OR, odds ratio. **Fig. S2**. A panel of scatterplots on the within-trial standard error of log OR for ‘old intervention versus placebo’ (axis *y*) against the within-trial log OR for that comparison (axis *x*) for each simulation scenario. The colour key indicates the magnitude of covariance between the within-trial standard error of log OR and within-trial log OR for that comparison. MOD, missing outcome data; OR, odds ratio. **Fig. S3**. Dot plots on the bias of posterior mean of NMA log OR for all pairwise comparisons under one-stage and two-stage PM approaches while accounting for low missing outcome data, the size of trials (small, moderate), the event frequency (low, frequent) and the extent of *τ*^2^ (small, substantial).

## Data Availability

The datasets generated and analysed during the current study are available online at https://github.com/LoukiaSpin/One-stage-vs-two-stage-PM-models.git.

## Abbreviations

CCC: Concordance correlation coefficient
CI: Confidence interval
CrI: Credible interval
IMOR: Informative missingness odds ratio
MAR: Missing at random
MOD: Missing outcome data
NMA: Network meta-analysis
OR: Odds ratio

## References

[1] Akl EA, Carrasco-Labra A, Brignardello-Petersen R, Neumann I, Johnston BC, Sun X (2015). Reporting, handling and assessing the risk of bias associated with missing participant data in systematic reviews: a methodological survey. BMJ Open.

[2] Kahale LA, Diab B, Brignardello-Petersen R, Agarwal A, Mustafa RA, Kwong J (2018). Systematic reviews do not adequately report or address missing outcome data in their analyses: a methodological survey. J Clin Epidemiol.

[3] Spineli LM, Pandis N, Salanti G (2015). Reporting and handling missing outcome data in mental health: a systematic review of Cochrane systematic reviews and meta-analyses. Res Synth Methods.

[4] Turner NL, Dias S, Ades AE, Welton NJ (2015). A Bayesian framework to account for uncertainty due to missing binary outcome data in pairwise meta-analysis. Stat Med.

[5] Spineli LM, Kalyvas C, Pateras K (2019). Participants’ outcomes gone missing within a network of interventions: Bayesian modeling strategies. Stat Med.

[6] Spineli LM (2019). An empirical comparison of Bayesian modelling strategies for missing binary outcome data in network meta-analysis. BMC Med Res Methodol.

[7] White IR, Higgins JPT, Wood AM (2008). Allowing for uncertainty due to missing data in meta-analysis--part 1: two-stage methods. Stat Med.

[8] Little RJA (1993). Pattern-mixture models for multivariate incomplete data. J Am Stat Assoc.

[9] Higgins JP, White IR, Wood AM (2008). Imputation methods for missing outcome data in meta-analysis of clinical trials. Clin Trials.

[10] Chaimani A, Mavridis D, Higgins JPT, Salanti G, White IR (2018). Allowing for informative missingness in aggregate data meta-analysis with continuous or binary outcomes: extensions to metamiss. Stata J.

[11] Bradburn MJ, Deeks JJ, Berlin JA, Localio AR (2007). Much ado about nothing: a comparison of the performance of meta-analytical methods with rare events. Stat Med.

[12] Sweeting MJ, Sutton AJ, Lambert PC (2004). What to add to nothing? Use and avoidance of continuity corrections in meta-analysis of sparse data. Stat Med.

[13] Davey J, Turner RM, Clarke MJ, Higgins JPT (2011). Characteristics of meta-analyses and their component studies in the Cochrane database of systematic reviews: a cross-sectional, descriptive analysis. BMC Med Res Methodol.

[14] Nikolakopoulou A, Chaimani A, Veroniki AA, Vasiliadis HS, Schmid CH, Salanti G (2014). Characteristics of networks of interventions: a description of a database of 186 published networks. PLoS One.

[15] Jackson D, White IR (2018). When should meta-analysis avoid making hidden normality assumptions?. Biom J.

[16] Hamza TH, van Houwelingen HC, Stijnen T (2008). The binomial distribution of meta-analysis was preferred to model within-study variability. J Clin Epidemiol.

[17] Stijnen T, Hamza TH, Özdemir P (2010). Random effects meta-analysis of event outcome in the framework of the generalized linear mixed model with applications in sparse data. Stat Med.

[18] Jackson D, Law M, Stijnen T, Viechtbauer W, White IR (2018). A comparison of seven random-effects models for meta-analyses that estimate the summary odds ratio. Stat Med.

[19] Seide SE, Jensen K, Kieser M (2020). A comparison of Bayesian and frequentist methods in random-effects network meta-analysis of binary data. Res Synth Methods.

[20] Spineli LM, Kalyvas C (2020). Comparison of exclusion, imputation and modelling of missing binary outcome data in frequentist network meta-analysis. BMC Med Res Methodol.

[21] Higgins JP, Whitehead A. Borrowing strength from external trials in a meta-analysis. Stat Med. 1996;15(24):2733–49. 10.1002/(SICI)1097-0258(19961230)15:24<2733::AID-SIM562>3.0.CO;2-0 8981683

[22] Salanti G (2012). Indirect and mixed-treatment comparison, network, or multiple-treatments meta-analysis: many names, many benefits, many concerns for the next generation evidence synthesis tool. Res Synth Methods.

[23] Lu G, Ades AE (2006). Assessing evidence inconsistency in mixed treatment comparisons. J Am Stat Assoc.

[24] Salanti G, Ades AE, Ioannidis JPA (2011). Graphical methods and numerical summaries for presenting results from multiple-treatment meta-analysis: an overview and tutorial. J Clin Epidemiol.

[25] Dias S, Sutton AJ, Ades AE, Welton NJ (2013). Evidence synthesis for decision making 2: a generalized linear modeling framework for pairwise and network meta-analysis of randomized controlled trials. Med Decis Mak.

[26] Sackett DL, Richardson WS, Rosenberg WM, Haynes RB (1997). Evidence-based medicine: how to practice and teach EBM.

[27] Su Y, Yajima M. R2jags: Using R to Run ‘JAGS’. R package version 0.5–7. 2015. https://cran.r-project.org/package=R2jags.

[28] R Core Team (2019). R: a language and environment for statistical computing.

[29] Lin LI (1989). A concordance correlation coefficient to evaluate reproducibility. Biometrics.

[30] Stevenson M. epiR: Tools for the Analysis of Epidemiological Data. R package version 1.0–15. 2020. https://cran.r-project.org/package=epiR.

[31] Wickham H (2009). ggplot2: Elegant Graphics for Data Analysis.

[32] Veroniki AA, Mavridis D, Higgins JPT, Salanti G (2014). Characteristics of a loop of evidence that affect detection and estimation of inconsistency: a simulation study. BMC Med Res Methodol.

[33] Turner RM, Jackson D, Wei Y, Thompson SG, Higgins JPT (2015). Predictive distributions for between-study heterogeneity and simple methods for their application in Bayesian meta-analysis. Stat Med.

[34] Hartung J, Knapp G (2001). A refined method for the meta-analysis of controlled clinical trials with binary outcome. Stat Med.

[35] White IR, Welton NJ, Wood AM, Ades AE, Higgins JPT (2008). Allowing for uncertainty due to missing data in meta-analysis--part 2: hierarchical models. Stat Med.

[36] White IR, Carpenter J, Horton NJ (2012). Including all individuals is not enough: lessons for intention-to-treat analysis. Clin Trials.

[37] Zhang Y, Akl EA, Schünemann HJ. Using systematic reviews in guideline development: the GRADE approach. Res Synth Methods. 2018. 10.1002/jrsm.1313. 10.1002/jrsm.1313 30006970

[38] Brignardello-Petersen R, Bonner A, Alexander PE, Siemieniuk RA, Furukawa TA, Rochwerg B (2018). Advances in the GRADE approach to rate the certainty in estimates from a network meta-analysis. J Clin Epidemiol.

[39] Chang B-H, Hoaglin DC (2017). Meta-analysis of odds ratios: current good practices. Med Care.

[40] Welton NJ, Phillippo DM, Owen R, Jones HJ, Dias S, Bujkiewicz S, Ades AE, Abrams KR. DSU Report. CHTE2020 Sources and Synthesis of Evidence; Update to Evidence Synthesis Methods. March 2020.

