# Supplementary material for: Pattern-mixture model in network meta-analysis of binary missing outcome data: one-stage or two-stage approach?
Source: BMC Med Res Methodol. 2021 Jan 7;21:12. doi: 10.1186/s12874-020-01205-6 (PMC7792003; doi:10.1186/s12874-020-01205-6)
Supplement: Supplementary file 1 — Additional file 1: Table S1. Comparison size and factors that affect within-trial normal approximation. Table S2. The posterior mean of residual deviance of each model per network. Table S3. Posterior mean (95% CrI) and bias (width of 95% CrI) for log OR (new versus old) under half-normal prior distribution on τ. Table S4. Posterior mean (95% CrI) and bias (width of 95% CrI) for log OR (new versus placebo) under half-normal prior distribution on τ. Table S5. Posterior mean (95% CrI) and bias (width of 95% CrI) for log OR (old versus placebo) under half-normal prior distribution on τ. Table S6. Posterior median (95% CrI) and bias (width of 95% CrI) for common τ2 under half-normal prior distribution on τ. Table S7. Posterior mean (95% CrI) and bias (width of 95% CrI) for log OR (new versus old intervention) under empirical prior distribution on τ. Table S8. Posterior mean (95% CrI) and bias (width of 95% CrI) for log OR (new vs placebo) under empirical prior distribution on τ. Table S9. Posterior mean (95% CrI) and bias (width of 95% CrI) for log OR (old versus placebo) under empirical prior distribution on τ. Fig. S1. A panel of scatterplots on the within-trial standard error of log OR for ‘new intervention versus placebo’ (axis y) against the within-trial log OR for that comparison (axis x) for each simulation scenario. The colour key indicates the magnitude of covariance between the within-trial standard error of log OR and within-trial log OR for that comparison. MOD, missing outcome data; OR, odds ratio. Fig. S2. A panel of scatterplots on the within-trial standard error of log OR for ‘old intervention versus placebo’ (axis y) against the within-trial log OR for that comparison (axis x) for each simulation scenario. The colour key indicates the magnitude of covariance between the within-trial standard error of log OR and within-trial log OR for that comparison. MOD, missing outcome data; OR, odds ratio. Fig. S3. Dot plots on the bias of posterior mean of NMA log [file 12874_2020_1205_MOESM1_ESM.docx]

**Model specification of random-effects network meta-analysis model for binary outcome**

For the one-stage and two-stage pattern-mixture (PM) approaches, we considered non-informative normal prior distributions with zero mean and variance equal to 10,000 on all location parameters, whereas empirical distributions tailored to the outcome and intervention-comparison type investigated in each network were assigned on $\tau^{2}$, as suggested by Turner et al. [1] to improve the estimation of the parameter in case of sparse networks and/ or rare events. A sensitivity analysis to different plausible prior distributions for $\tau^{2}$ should be opted systematically by the authors of systematic reviews to investigate the sensitivity of the primary analysis results. We did not perform such a sensitivity analysis as it is out of the scope of the present study.

For each network, we obtained the posterior mean of residual deviance to investigate whether each model fits the data satisfactorily [2, 3]. A lack of fit is inferred when the posterior mean of residual deviance is greater than the number of observed independent points [2, 3]. Under the one-stage PM model, each trial-arm corresponds to a data-point and contributes one independent data-point to the posterior mean deviance [2, 3]. Under the two-stage PM model, each treatment contrast with the baseline arm of the trial corresponds to a data-point and contributes one independent data-point to the posterior mean deviance [2, 3].

We used three parallel chains of different initial values and thinning equal to 10 for 100,000 updates and a burn-in of 10,000 Markov chain Monte Carlo samples. Convergence assessment was based on the Gelman–Rubin convergence diagnostic, $\hat{R}$, and inspections of trace plots [4]. We excluded results on parameters that corresponded to Gelman–Rubin $\hat{R}$ larger than 1.1, as convergence for those parameters was not achieved, and therefore, the corresponding posterior distributions could not be trusted. Specifically, convergence failed for one within-trial log OR and one NMA log OR from the one-stage PM approach for having implausibly large posterior standard deviation (both equal to 26).

**References**

1. Turner NL, Dias S, Ades AE, Welton NJ. A Bayesian framework to account for uncertainty due to missing binary outcome data in pairwise meta-analysis. Stat Med. 2015;34(12):2062-2080. doi:10.1002/sim.6475.
2. Spiegelhalter DJ, Best NG, Carlin BP, van der Linde A. Bayes- ian measures of model complexity and fit. J Roy Stat Soc B. 2002; 64(4):583–616.
3. Dias S, Sutton AJ, Ades AE, Welton NJ. Evidence synthesis for decision making 2: a generalized linear modeling framework for pairwise and network meta-analysis of randomized controlled trials. Med Decis Mak. 2013;33(5):607–17.
4. Gelman A, Rubin D. Inference from Iterative Simulation Using Multiple Sequences. Stat Sci. 1992;7(4):457-472

**Data-generating model of Hartung and Knapp**

First, we generated *initial* event risks for the experimental arm $p_{i,k}^{E,0}$ with $k=New,Old$ as a function of pre-specified *initial* event risks in the control arm $p_{i,k}^{C,0}$ with $k=Old,Placebo$ and underlying log odds ratio $\mu_{kl}$ ($k=New,Old,$ $l=Old,Placebo$ and $k\neq l$) via the equations below [1]:

$$p_{i,k}^{E,0}=\frac{p_{i,P}^{C,0}\cdot exp\left( \mu_{kP} \right)}{1-p_{i,P}^{C,0}+p_{i,P}^{C,0}\cdot exp\left( \mu_{kP} \right)}, k=New,Old$$

$$p_{i,N}^{E,0}=\frac{p_{i,O}^{C,0}\cdot exp\left( \mu_{NO} \right)}{1-p_{i,O}^{C,0}+p_{i,O}^{C,0}\cdot exp\left( \mu_{NO} \right)}$$

for the placebo- and old-controlled trials, respectively. We used information from our collection with networks from several health-related fields to define the *initial* event risks for the control arms [2,3]. Specifically, we generated the *initial* event risks for the control arms from the following uniform distributions that correspond to low and frequent events:

Low events

$p_{i,P}^{C,0}\sim U\left( 0.05, 0.09 \right)$ and $p_{i,O}^{C,0}\sim U\left( 0.10, 0.15 \right)$

Frequent events

$p_{i,P}^{C,0}\sim U\left( 0.27, 0.40 \right)$ and $p_{i,O}^{C,0}\sim U\left( 0.63, 0.76 \right)$

for the placebo- and old-controlled trials, respectively. Then, we calculated the underlying log odds for each arm in every trial as follows:

$${logit}_{i,k}^{E,0}=log\left( \frac{p_{i,k}^{E,0}}{1-p_{i,k}^{E,0}} \right), k=New,Old$$

$${logit}_{i,k}^{C,0}=log\left( \frac{p_{i,k}^{C,0}}{1-p_{i,k}^{C,0}} \right), k=Old,Placebo$$

for the experimental and control arm, respectively.

We incorporated the between-trial variance ($\tau^{2}$) in the following normal distributions to generate log odds for each arm in every trial, while assuming smaller variability in the log odds for placebo but equal in the log odds for active arms:

Placebo-controlled

${logit}_{i,k}^{E}\sim N\left( {logit}_{i,k}^{E,0},\frac{{2\tau}^{2}}{3} \right), k=New,Old$and${logit}_{i,k}^{C}\sim N\left( {logit}_{i,k}^{C,0},\frac{\tau^{2}}{3} \right), k=Old,Placebo$

Old-controlled

$${logit}_{i,N}^{C}\sim N\left( {logit}_{i,k}^{C,0},\frac{\tau^{2}}{2} \right) \mathrm{and} {logit}_{i,O}^{C}\sim N\left( {logit}_{i,k}^{C,0},\frac{\tau^{2}}{2} \right)$$

We selected the predictive log-normal distributions *L*$N\left( -3.95, {1.34}^{2} \right)$ (median: 0.02, 95% prior interval: 0.001 – 0.26) and *L*$N\left( -2.56, {1.74}^{2} \right)$ (median: 0.07, 95% prior interval: 0.002 – 2.67) to indicate small and substantial true $\tau^{2}$, respectively. These predictive distributions referred to the expected $\tau^{2}$ in a future meta-analysis for all-cause mortality and a generic healthcare setting, respectively [4].

Then, we back-calculated the underlying event risks in each arm of every trial for the experimental and control arm as follows:

$$p_{i,k}^{E}=\frac{1}{1+exp\left( -{logit}_{i,k}^{E} \right)}, k=New,Old$$

$$p_{i,k}^{C}=\frac{1}{1+exp\left( {-logit}_{i,k}^{C} \right)}, k=Old,Placebo$$

**References**

1. Hartung J, Knapp G. A refined method for the meta-analysis of controlled clinical trials with binary outcome. *Stat Med* 2001;20(24):3875-3889.
2. Spineli LM, Kalyvas C, Pateras K. Participants' outcomes gone missing within a network of interventions: Bayesian modeling strategies. *Stat Med* 2019;38(20):3861-3879.
3. Spineli LM. An empirical comparison of Bayesian modelling strategies for missing binary outcome data in network meta-analysis. *BMC Med Res Methodol* 2019;19(1):86.
4. Turner RM, Jackson D, Wei Y, Thompson SG, Higgins JPT. Predictive distributions for between-study heterogeneity and simple methods for their application in Bayesian meta-analysis. *Stat Med* 2015;34(6):984-998.

**TABLES**

**Table S1. Comparison size and factors that affect within-trial normal approximation**

| **Network^1^** | **% comparisons with** | | | **Trial sample size^2^** | **event risk across arms^2^** | **No. trials with at least one zero-cell** |
| --- | --- | --- | --- | --- | --- | --- |
|  | **1 trial** | **2 trials** | **> 2 trials** |  |  |  |
| 1 | 41% | 20% | 39% | 237 (112, 300)  (22, 708) | 0.62 (0.51, 0.69) (0.10, 0.95) | 0 |
| 2 | 25% | 25% | 50% | 235 (212, 391)  (86, 1037) | 0.74 (0.58, 0.88) (0.44, 0.98) | 0 |
| 3 | 40% | 20% | 40% | 408 (267, 653)  (16, 1829) | 0.65 (0.55, 0.81) (0.23, 1.00) | 1 |
| 4 | 50% | 0% | 50% | 389 (356, 446) (266, 471) | 0.73 (0.68, 0.79) (0.65, 0.91) | 0 |
| 5 | 20% | 80% | 0% | 203 (128, 290)  (74, 322) | 0.45 (0.31, 0.57) (0.16, 0.83) | 0 |
| 6 | 56% | 22% | 22% | 474 (312, 850) (177, 1505) | 0.48 (0.27, 0.56) (0.14, 0.75) | 0 |
| 7 | 9% | 9% | 82% | 199 (83, 295) (23, 694) | 0.24 (0.14, 0.44) (0.00, 0.75) | 1 |
| 8 | 50% | 0% | 50% | 352 (284, 700) (154, 1897) | 0.92 (0.86, 0.93) (0.72, 0.96) | 0 |
| 9 | 40% | 40% | 20% | 381 (222, 603) (117, 716) | 0.58 (0.50, 0.71) (0.40, 0.95) | 0 |
| 10 | 57% | 43% | 0% | 303 (186, 408)  (89, 638) | 0.65 (0.44, 0.77) (0.33, 0.96) | 0 |
| 11 | 70% | 10% | 20% | 158 (51, 405)  (40, 1171) | 0.40 (0.33, 0.56) (0.00, 0.83) | 1 |
| 12 | 33% | 0% | 67% | 402 (283, 511) (144, 1127) | 0.29 (0.21, 0.45) (0.12, 0.70) | 0 |
| 13 | 0% | 0% | 100% | 349 (304, 419) (252, 538) | 0.31 (0.22, 0.44) (0.13, 0.59) | 0 |
| 14 | 50% | 25% | 25% | 48 (39, 61) (12, 296) | 0.47 (0.23, 0.72) (0.09, 1.00) | 1 |
| 15 | 80% | 20% | 0% | 497 (437, 601) (319, 846) | 0.82 (0.66, 0.95) (0.58, 0.98) | 0 |
| 16 | 75% | 0% | 25% | 1161 (247, 2839) (137, 5241) | 0.94 (0.92, 0.98) (0.89, 0.99) | 0 |
| 17 | 73% | 0% | 27% | 1053 (784, 6918) (75, 18201) | 0.95 (0.92, 0.97) (0.57, 1.00) | 1 |
| 18 | 74% | 16% | 10% | 85 (50, 245) (30, 635) | 0.62 (0.42, 0.71) (0.00, 0.96) | 1 |
| 19 | 0% | 75% | 25% | 187 (151, 289) (105, 516) | 0.66 (0.50, 0.82) (0.23, 0.99) | 0 |
| 20 | 77% | 15% | 8% | 690 (401, 795) (106, 883) | 0.86 (0.77, 0.89) (0.35, 0.93) | 0 |
| 21 | 100% | 0% | 0% | 488 (346, 756) (234, 859) | 0.70 (0.41, 0.83) (0.17, 0.87) | 0 |
| 22 | 38% | 38% | 25% | 314 (130, 520)  (71, 4128) | 0.95 (0.86, 0.98) (0.28, 1.00) | 4 |
| 23 | 50% | 50% | 0% | 4140 (2788,5254) (2540, 8240) | 0.98 (0.97, 0.98) (0.96, 0.98) | 0 |
| 24 | 50% | 20% | 30% | 78 (44, 98) (22, 318) | 0.79 (0.69, 0.90) (0.29, 1.00) | 2 |
| 25 | 19% | 25% | 56% | 163 (98, 329) (40, 1385) | 0.60 (0.51, 0.70) (0.17, 0.90) | 0 |
| 26 | 75% | 25% | 0% | 503 (304, 600) (140, 600) | 0.92 (0.88, 0.95) (0.75, 0.99) | 0 |
| 27 | 56% | 28% | 17% | 570 (119, 1108) (40, 1512) | 0.49 (0.36, 0.57) (0.00, 0.89) | 1 |
| 28 | 20% | 0% | 80% | 339 (291, 424)  (90, 905) | 0.43 (0.36, 0.57) (0.25, 0.91) | 0 |
| 29 | 83% | 17% | 0% | 563 (304, 783)  (79, 883) | 0.80 (0.76, 0.84) (0.71, 0.88) | 0 |

^1^We have highlighted in grey the ‘susceptible’ networks with at least one trial with total sample size less than 50 participants and/or observed event risk less than 5%.

^2^Results are presented as median, interquartile range (1rst, 3rd quartile) and range (minimum, maximum).

**References of analysed networks**

1. Cipriani A, Furukawa TA, Salanti G, Geddes JR, Higgins JP, Churchill R, et al. Comparative efficacy and acceptability of 12 new-generation antidepressants: a multiple-treatments meta-analysis. Lancet. 2009;373:746-58.
2. Edwards SJ, Clarke MJ, Wordsworth S, Welton NJ. Carbapenems versus other beta-lactams in the treatment of hospitalised patients with infection: a mixed treatment comparison. Curr Med Res Opin. 2009;25:251-61.
3. Baker WL, Baker EL, Coleman CI. Pharmacologic treatments for chronic obstructive pulmonary disease: a mixed-treatment comparison meta-analysis. Pharmacotherapy. 2009;29:891-905.
4. Burch J, Paulden M, Conti S, Stock C, Corbett M, Welton NJ, et al. Antiviral drugs for the treatment of influenza: a systematic review and economic evaluation. Health Technol Assess. 2009;13:1-265, iii-iv.
5. Uthman OA, Abdulmalik J. Comparative efficacy and acceptability of pharmacotherapeutic agents for anxiety disorders in children and adolescents: a mixed treatment comparison meta-analysis. Curr Med Res Opin. 2010;26:53-9.
6. Bottomley JM, Taylor RS, Ryttov J. The effectiveness of two-compound formulation calcipotriol and betamethasone dipropionate gel in the treatment of moderately severe scalp psoriasis: a systematic review of direct and indirect evidence. Curr Med Res Opin. 2011;27:251-68.
7. Costa J, Fareleira F, Ascenção R, Borges M, Sampaio C, Vaz-Carneiro A. Clinical comparability of the new antiepileptic drugs in refractory partial epilepsy: a systematic review and meta-analysis. Epilepsia. 2011;52:1280-91.
8. Makani H, Bangalore S, Romero J, Wever-Pinzon O, Messerli FH. Effect of renin-angiotensin system blockade on calcium channel blocker-associated peripheral edema. Am J Med. 2011;124:128-35.
9. Virgili G, Novielli N, Menchini F, Murro V, Giacomelli G. Pharmacological treatments for neovascular age-related macular degeneration: can mixed treatment comparison meta-analysis be useful? Curr Drug Targets. 2011;12:212-20.
10. Gallego-Galisteo M, Villa-Rubio A, Alegre-del Rey E, Márquez-Fernández E, Ramos-Báez JJ. Indirect comparison of biological treatments in refractory rheumatoid arthritis. J Clin Pharm Ther. 2012;37:301-7.
11. Filippini G, Del Giovane C, Vacchi L, D'Amico R, Di Pietrantonj C, Beecher D, et al. Immunomodulators and immunosuppressants for multiple sclerosis: a network meta-analysis. Cochrane Database Syst Rev. 2013;CD008933.
12. Gao L, Xia L, Zhao FL, Li SC. Clinical efficacy and safety of the newer antiepileptic drugs as adjunctive treatment in adults with refractory partial-onset epilepsy: a meta-analysis of randomized placebo-controlled trials. Epilepsy Res. 2013;103:31-44.
13. Khan N, Shah D, Tongbram V, Verdian L, Hawkins N. The efficacy and tolerability of perampanel and other recently approved anti-epileptic drugs for the treatment of refractory partial onset seizure: a systematic review and Bayesian network meta-analysis. Curr Med Res Opin. 2013;29:1001-13.
14. Liu J, Dong J, Wang L, Su Y, Yan P, Sun S. Comparative efficacy and acceptability of antidepressants in Parkinson's disease: a network meta-analysis. PLoS One. 2013;8:e76651.
15. Mealing S, Barcena L, Hawkins N, Clark J, Eaton V, Hirji I, et al. The relative efficacy of imatinib, dasatinib and nilotinib for newly diagnosed chronic myeloid leukemia: a systematic review and network meta-analysis. Exp Hematol Oncol. 2013;2:5.
16. Wu MS, Tan SC, Xiong T. Indirect comparison of randomised controlled trials: comparative efficacy of dexlansoprazole vs. esomeprazole in the treatment of gastro-oesophageal reflux disease. Aliment Pharmacol Ther. 2013;38:190-201.
17. Dogliotti A, Paolasso E, Giugliano RP. Current and new oral antithrombotics in non-valvular atrial fibrillation: a network meta-analysis of 79 808 patients. Heart. 2014;100:396-405.
18. Kriston L, von Wolff A, Westphal A, Hölzel LP, Härter M. Efficacy and acceptability of acute treatments for persistent depressive disorder: a network meta-analysis. Depress Anxiety. 2014;31:621-30.
19. Roskell NS, Setyawan J, Zimovetz EA, Hodgkins P. Systematic evidence synthesis of treatments for ADHD in children and adolescents: indirect treatment comparisons of lisdexamfetamine with methylphenidate and atomoxetine. Curr Med Res Opin. 2014;30:1673-85.
20. Patel DA, Snedecor SJ, Tang WY, Sudharshan L, Lim JW, Cuffe R, et al. 48-week efficacy and safety of dolutegravir relative to commonly used third agents in treatment-naive HIV-1-infected patients: a systematic review and network meta-analysis. PLoS One. 2014;9:e105653.
21. Fournier M, Germe M, Theobald K, Scholz GH, Lehmacher W. Indirect comparison of lixisenatide versus neutral protamine Hagedorn insulin as add-on to metformin and sulphonylurea in patients with type 2 diabetes mellitus. Ger Med Sci. 2014;12:Doc14.
22. Palmer SC, Saglimbene V, Mavridis D, Salanti G, Craig JC, Tonelli M, et al. Erythropoiesis-stimulating agents for anaemia in adults with chronic kidney disease: a network meta-analysis. Cochrane Database Syst Rev. 2014;CD010590.
23. Mantha S, Ansell J. Indirect comparison of dabigatran, rivaroxaban, apixaban and edoxaban for the treatment of acute venous thromboembolism. J Thromb Thrombolysis. 2015;39:155-65.
24. Singh S, Garg SK, Pardi DS, Wang Z, Murad MH, Loftus EV Jr. Comparative efficacy of pharmacologic interventions in preventing relapse of Crohn's disease after surgery: a systematic review and network meta-analysis. Gastroenterology. 2015;148:64-76.e2.
25. Linde K, Kriston L, Rücker G, Jamil S, Schumann I, Meissner K, et al. Efficacy and acceptability of pharmacological treatments for depressive disorders in primary care: systematic review and network meta-analysis. Ann Fam Med. 2015;13:69-79.
26. Bow EJ, Vanness DJ, Slavin M, Cordonnier C, Cornely OA, Marks D, et al. Systematic review and mixed treatment comparison meta-analysis of randomized clinical trials of primary oral antifungal prophylaxis in allogeneic hematopoietic cell transplant recipients. BMC Infect Dis. 2015;15:128.
27. Tramacere I, Del Giovane C, Salanti G, D'Amico R, Filippini G. Immunomodulators and immunosuppressants for relapsing-remitting multiple sclerosis: a network meta-analysis. Cochrane Database Syst Rev. 2015;CD011381.
28. Miligkos M, Papamichael K, Vande Casteele N, Mantzaris GJ, Gils A, Levesque BG, et al. Efficacy and Safety Profile of Anti-tumor Necrosis Factor-α Versus Anti-integrin Agents for the Treatment of Crohn's Disease: A Network Meta-analysis of Indirect Comparisons. Clin Ther. 2016;38:1342-1358.e6.
29. Vieira MC, Kumar RN, Jansen JP. Comparative effectiveness of efavirenz, protease inhibitors, and raltegravir-based regimens as first-line treatment for HIV-infected adults: a mixed treatment comparison. HIV Clin Trials. 2011;12:175-89.

**Table S2.** **The posterior mean of residual deviance of each model per network**

| **Network** | **One-stage pattern-mixture model** | | **Two-stage pattern-mixture model** | |
| --- | --- | --- | --- | --- |
|  | ${\bar{\boldsymbol{D}}}_{\boldsymbol{res}}$ | **Number of points** | ${\bar{\boldsymbol{D}}}_{\boldsymbol{res}}$ | **Number of points** |
| 1 | 204.10 | 209 | 97.19 | 105 |
| 2 | 26.99 | 30 | 11.70 | 15 |
| 3 | **51.42** | **50** | 23.67 | 29 |
| 4 | 11.35 | 12 | 5.20 | 6 |
| 5 | **19.23** | **18** | **10.27** | **9** |
| 6 | **22.07** | **21** | **13.24** | **12** |
| 7 | 119.21 | 119 | 56.91 | 60 |
| 8 | 9.37 | 12 | 3.30 | 6 |
| 9 | 19.17 | 20 | 8.87 | 10 |
| 10 | 20.34 | 20 | 9.95 | 10 |
| 11 | **35.10*** | **32** | **19.21*** | **16** |
| 12 | 30.50 | 30 | 15.73 | 15 |
| 13 | 24.49 | 24 | 12.36 | 12 |
| 14 | 25.10 | 25 | **15.42** | **14** |
| 15 | 8.72 | 9 | 4.58 | 5 |
| 16 | **9.88** | **9** | **5.92** | **5** |
| 17 | 33.00 | 36 | 16.60 | 20 |
| 18 | **74.78** | **73** | 36.67 | 39 |
| 19 | **31.65*** | **29** | **19.73*** | **15** |
| 20 | 29.55 | 31 | 14.25 | 16 |
| 21 | 10.08 | 10 | 5.01 | 5 |
| 22 | 30.97 | 32 | 15.09 | 16 |
| 23 | 11.98 | 12 | 5.96 | 6 |
| 24 | **41.93*** | **39** | 19.58 | 20 |
| 25 | 107.77 | 107 | 56.35 | 57 |
| 26 | 10.24 | 10 | 5.13 | 5 |
| 27 | **63.82*** | **57** | **35.61*** | **29** |
| 28 | 26.30 | 26 | 13.20 | 13 |
| 29 | 12.99 | 14 | 5.99 | 7 |

A lack of fit (posterior mean of residual deviance, $\bar{D}_{res}$, is greater than the number of observed independent points) is indicated in bold with an asterisk. Possible lack of fit is indicated in bold.

The references of the networks can be found after Table S1.

| **Half-normal prior distribution on common between-trial standard deviation** |
| --- |

**Table S3. Posterior mean (95% CrI) and bias (width of 95% CrI) for log OR (new versus old)**

| small $\tau^{2}$ | | moderate MOD | | large MOD | |
| --- | --- | --- | --- | --- | --- |
| trial size | frequency | one-stage | two-stage | one-stage | two-stage |
| small | low | 0.42 (-2.28, 3.26)  0.13 (5.54) | 0.32 (-1.73, 2.33)  0.03 (4.06) | 0.54 (-2.97, 4.61)  0.25 (7.59) | 0.32 (-2.02, 2.64)  0.03 (4.65) |
| moderate | low | 0.37 (-0.68, 1.46)  0.08 (2.15) | 0.37 (-0.65, 1.41)  0.08 (2.06) | 0.39 (-1.14, 2.03)  0.10 (3.17) | 0.37 (-1.09, 1.89)  0.08 (2.98) |
| small | frequent | 0.33 (-1.07, 1.76)  0.04 (2.83) | 0.32 (-1.04, 1.69)  0.03 (2.73) | 0.40 (-1.24, 2.08)  0.11 (3.32) | 0.38 (-1.22, 2.00)  0.09 (3.21) |
| moderate | frequent | 0.33 (-0.35, 1.02)  0.04 (1.37) | 0.33 (-0.36, 1.03)  0.04 (1.39) | 0.36 (-0.55, 1.29)  0.07 (1.84) | 0.36 (-0.59, 1.34)  0.07 (1.93) |
|  | | | | | |
| substantial $\tau^{2}$ | | moderate MOD | | large MOD | |
| trial size | frequency | one-stage | two-stage | one-stage | two-stage |
| small | low | 0.43 (-2.42, 3.63)  0.14 (6.06) | 0.33 (-1.77, 2.45)  0.04 (4.22) | 0.50 (-3.06, 4.88)  0.22 (7.93) | 0.29 (-2.05, 2.65)  0.01 (4.70) |
| moderate | low | 0.38 (-1.04, 1.81)  0.09 (2.85) | 0.38 (-0.93, 1.70)  0.09 (2.63) | 0.44 (-1.38, 2.36) 0.15 (3.74) | 0.41 (-1.26, 2.12)  0.12 (3.38) |
| small | frequent | 0.35 (-1.26, 2.05)  0.07 (3.31) | 0.34 (-1.20, 1.91)  0.05 (3.11) | 0.40 (-1.55, 2.32)  0.11 (3.88) | 0.37 (-1.46, 2.17)  0.08 (3.63) |
| moderate | frequent | 0.32 (-0.73, 1.39)  0.03 (2.12) | 0.32 (-0.74, 1.40)  0.03 (2.14) | 0.36 (-0.93, 1.60)  0.07 (2.52) | 0.36 (-0.96, 1.64)  0.08 (2.60) |

Posterior mean and 95% CrI are provided in the greyed area, followed by bias and width of 95% CrI (in parenthesis) in the white area.

**Table S4. Posterior mean (95% CrI) and bias (width of 95% CrI) for log OR (new versus placebo)**

| small $\tau^{2}$ | | moderate MOD | | large MOD | |
| --- | --- | --- | --- | --- | --- |
| trial size | frequency | one-stage | two-stage | one-stage | two-stage |
| small | low | 0.53 (-1.71, 3.11)  -0.16 (4.82) | 0.37 (-1.38, 2.13)  -0.32 (3.51) | -0.04 (-3.43, 3.36)  -0.73 (6.78) | -0.05 (-2.09, 1.95)  -0.75 (4.04) |
| moderate | low | 0.44 (-0.49, 1.40)  -0.25 (1.89) | 0.44 (-0.46, 1.36)  -0.25 (1.81) | -0.10 (-1.55, 1.81)  -0.79 (2.73) | -0.09 (-1.44, 1.13)  -0.78 (2.57) |
| small | frequent | 0.51 (-0.68, 1.71)  -0.18 (2.39) | 0.49 (-0.67, 1.64)  -0.20 (2.31) | 0.14 (-1.25, 1.55)  -0.55 (2.80) | 0.13 (-1.23, 1.50)  -0.56 (2.72) |
| moderate | frequent | 0.49 (-0.10, 1.05)  -0.20 (1.15) | 0.50 (-0.10, 1.07)  -0.19 (1.17) | 0.13 (-0.69, 0.90)  -0.57 (1.60) | 0.12 (-0.73, 0.94)  -0.57 (1.67) |
|  | | | | | |
| substantial $\tau^{2}$ | | moderate MOD | | large MOD | |
| trial size | frequency | one-stage | two-stage | one-stage | two-stage |
| small | low | 0.49 (-2.11, 3.32)  -0.20 (5.43) | 0.34 (-1.53, 2.19)  -0.35 (3.72) | -0.13 (-3.53, 3.21)  -0.82 (6.75) | -0.10 (-2.14, 1.92)  -0.79 (4.06) |
| moderate | low | 0.42 (-0.89, 1.61)  -0.28 (2.50) | 0.42 (-0.79, 1.54)  -0.27 (2.33) | -0.11 (-1.85, 1.41) -0.81 (3.26) | -0.10 (-1.65, 1.32)  -0.79 (2.97) |
| small | frequent | 0.48 (-0.91, 1.89)  -0.21 (2.80) | 0.46 (-0.87, 1.79)  -0.23 (2.66) | 0.06 (-1.67, 1.69)  -0.63 (3.36) | 0.05 (-1.56, 1.59)  -0.64 (3.15) |
| moderate | frequent | 0.46 (-0.58, 1.36)  -0.23 (1.94) | 0.47 (-0.57, 1.37)  -0.22 (1.95) | 0.10 (-1.17, 1.11)  -0.59 (2.28) | 0.10 (-1.20, 1.14)  -0.59 (2.34) |

Posterior mean and 95% CrI are provided in the greyed area, followed by bias and width of 95% CrI (in parenthesis) in the white area.

**Table S5. Posterior mean (95% CrI) and bias (width of 95% CrI) for log OR (old versus placebo)**

| small $\tau^{2}$ | | moderate MOD | | large MOD | |
| --- | --- | --- | --- | --- | --- |
| trial size | frequency | one-stage | two-stage | one-stage | two-stage |
| small | low | 0.12 (-2.12, 2.51)  -0.29 (4.62) | 0.06 (-1.56, 1.69)  -0.35 (3.25) | -0.57 (-4.06, 2.28)  -0.97 (6.34) | -0.38 (-2.20, 1.46)  -0.78 (3.66) |
| moderate | low | 0.07 (-0.83, 0.95)  -0.33 (1.78) | 0.08 (-0.79, 0.92)  -0.33 (1.71) | -0.49 (-1.97, 0.73)  -0.90 (2.70) | -0.46 (-1.78, 0.70)  -0.86 (2.48) |
| small | frequent | 0.18 (-0.90, 1.25)  -0.23 (2.15) | 0.17 (-0.87, 1.20)  -0.23 (2.08) | -0.25 (-1.60, 1.03)  -0.66 (2.64) | -0.24 (-1.54, 1.01)  -0.65 (2.54) |
| moderate | frequent | 0.16 (-0.41, 0.69)  -0.24 (1.10) | 0.17 (-0.41, 0.71)  -0.24 (1.11) | -0.23 (-1.01, 0.50)  -0.64 (1.52) | -0.24 (-1.05, 0.53)  -0.64 (1.58) |
|  | | | | | |
| substantial $\tau^{2}$ | | moderate MOD | | large MOD | |
| trial size | frequency | one-stage | two-stage | one-stage | two-stage |
| small | low | 0.07 (-2.56, 2.60)  -0.34 (5.16) | 0.01 (-1.72, 1.71)  -0.39 (3.43) | -0.63 (-4.46, 2.46)  -1.04 (6.92) | -0.40 (-2.26, 1.47)  -0.80 (3.73) |
| moderate | low | 0.03 (-1.28, 1.22)  -0.37 (2.49) | 0.04 (-1.15, 1.13)  -0.37 (2.28) | -0.55 (-2.32, 0.89) -0.96 (3.21) | -0.51 (-1.99, 0.83)  -0.92 (2.82) |
| small | frequent | 0.14 (-1.36, 1.44)  -0.27 (2.79) | 0.13 (-1.24, 1.36)  -0.27 (2.60) | -0.33 (-1.96, 1.15)  -0.73 (3.11) | -0.31 (-1.80, 1.09)  -0.72 (2.90) |
| moderate | frequent | 0.14 (-0.86, 1.00)  -0.26 (1.86) | 0.15 (-0.86, 1.01)  -0.25 (1.87) | -0.25 (-1.40, 0.70)  -0.66 (2.11) | -0.26 (-1.43, 0.73)  -0.66 (2.15) |

Posterior mean and 95% CrI are provided in the greyed area, followed by bias and width of 95% CrI (in parenthesis) in the white area.

**Table S6. Posterior median (95% CrI) and bias (width of 95% CrI) for common** $\boldsymbol{\tau}^{\boldsymbol{2}}$

| small $\tau^{2}$ | | moderate MOD | | large MOD | |
| --- | --- | --- | --- | --- | --- |
| trial size | frequency | one-stage | two-stage | one-stage | two-stage |
| small | low | 0.37 (5$\times$10^-4^, 4.54)  0.35 (4.54) | 0.19 (4$\times$10^-4^, 2.42)  0.17 (2.42) | 0.47 (6$\times$10^-4^, 5.26)  0.45 (5.26) | 0.21 (4$\times$10^-4^, 2.57)  0.19 (2.57) |
| moderate | low | 0.09 (1$\times$10^-4^, 1.27)  0.07 (1.27) | 0.08 (2$\times$10^-4^, 1.15)  0.06 (1.15) | 0.13 (2$\times$10^-4^, 1.91)  0.11 (1.90) | 0.11 (2$\times$10^-4^, 1.60)  0.09 (1.60) |
| small | frequent | 0.13 (2$\times$10^-4^, 1.78)  0.11 (1.78) | 0.12 (3$\times$10^-4^, 1.59)  0.10 (1.59) | 0.15 (2$\times$10^-4^, 2.08)  0.14 (2.08) | 0.14 (3$\times$10^-4^, 1.83)  0.12 (1.83) |
| moderate | frequent | 0.03 (4$\times$10^-5^, 0.45)  0.01 (0.45) | 0.03 (6$\times$10^-5^, 0.46)  0.01 (0.46) | 0.04 (5$\times$10^-5^, 0.64)  0.02 (0.63) | 0.04 (9$\times$10^-5^, 0.68)  0.02 (0.68) |
|  | | | | | |
| substantial $\tau^{2}$ | | moderate MOD | | large MOD | |
| trial size | frequency | one-stage | two-stage | one-stage | two-stage |
| small | low | 0.40 (6$\times$10^-4^, 4.62)  0.32 (4.62) | 0.20 (4$\times$10^-4^, 2.44)  0.12 (2.44) | 0.49 (7$\times$10^-4^, 5.44)  0.41 (5.44) | 0.21 (5$\times$10^-4^, 2.60)  0.14 (2.60) |
| moderate | low | 0.12 (2$\times$10^-4^, 1.82)  0.04 (1.81) | 0.11 (2$\times$10^-4^, 1.53)  0.03 (1.53) | 0.16 (2$\times$10^-4^, 2.29)  0.08 (2.29) | 0.13 (3$\times$10^-4^, 1.77)  0.05 (1.77) |
| small | frequent | 0.16 (2$\times$10^-4^, 2.13)  0.08 (2.12) | 0.14 (3$\times$10^-4^, 1.81)  0.06 (1.81) | 0.18 (2$\times$10^-4^, 2.46)  0.11 (2.46) | 0.15 (3$\times$10^-4^, 2.03)  0.08 (2.03) |
| moderate | frequent | 0.05 (6$\times$10^-5^, 0.88)  -0.03 (0.88) | 0.05 (9$\times$10^-5^, 0.87)  -0.03 (0.87) | 0.05(6$\times$10^-5^, 0.97)  -0.03 (0.97) | 0.05 (1$\times$10^-4^, 0.97)  -0.03 (0.97) |

Posterior median and 95% CrI are provided in the greyed area, followed by bias and width of 95% CrI (in parenthesis) in the white area.

| **Empirical prior distribution on common between-trial variance** |
| --- |

**Table S7. Posterior mean (95% CrI) and bias (width of 95% CrI) for log OR (new versus old intervention)**

| small $\tau^{2}$ | | moderate MOD | | large MOD | |
| --- | --- | --- | --- | --- | --- |
| trial size | frequency | one-stage | two-stage | one-stage | two-stage |
| small | low | 0.42 (-2.04, 3.09)  0.13 (5.13) | 0.32 (-1.63, 2.23)  0.03 (3.86) | 0.52 (-2.65, 4.20)  0.23 (6.85) | 0.32 (-1.92, 2.53)  0.03 (4.45) |
| moderate | low | 0.37 (-0.62, 1.39)  0.08 (2.01) | 0.37 (-0.60, 1.35)  0.08 (1.95) | 0.39 (-1.06, 1.92)  0.10 (2.98) | 0.36 (-1.02, 1.81)  0.08 (2.83) |
| small | frequent | 0.33 (-0.98, 1.67)  0.05 (2.65) | 0.32 (-0.97, 1.61)  0.03 (2.57) | 0.40 (-1.15, 1.97)  0.11 (3.12) | 0.38 (-1.14, 1.91)  0.09 (3.05) |
| moderate | frequent | 0.33 (-0.34, 1.02)  0.04 (1.36) | 0.33 (-0.36, 1.03)  0.04 (1.38) | 0.36 (-0.53, 1.27)  0.07 (1.80) | 0.36 (-0.57, 1.32)  0.07 (1.89) |
|  | | | | | |
| substantial $\tau^{2}$ | | moderate MOD | | large MOD | |
| trial size | frequency | one-stage | two-stage | one-stage | two-stage |
| small | low | 0.42 (-2.13, 3.35)  0.13 (5.49) | 0.33 (-1.67, 2.36)  0.04 (4.03) | 0.48 (-2.72, 4.46)  0.19 (7.18) | 0.29 (-1.95, 2.55)  0.01 (4.50) |
| moderate | low | 0.38 (-0.94, 1.71)  0.09 (2.65) | 0.38 (-0.84, 1.62)  0.09 (2.47) | 0.44 (-1.27, 2.21) 0.15 (3.48) | 0.41 (-1.18, 2.03)  0.12 (3.21) |
| small | frequent | 0.35 (-1.16, 1.94)  0.07 (3.10) | 0.34 (-1.11, 1.82)  0.05 (2.93) | 0.39 (-1.43, 2.19)  0.11 (3.63) | 0.37 (-1.36, 2.07)  0.08 (3.44) |
| moderate | frequent | 0.32 (-0.70, 1.37)  0.03 (2.07) | 0.32 (-0.71, 1.37)  0.03 (2.08) | 0.36 (-0.90, 1.57)  0.07 (2.46) | 0.36 (-0.93, 1.61)  0.08 (2.55) |

Posterior mean and 95% CrI are provided in the greyed area, followed by bias and width of 95% CrI in parenthesis) in the white area.

**Table S8. Posterior mean (95% CrI) and bias (width of 95% CrI) for log OR (new *vs* placebo)**

| small $\tau^{2}$ | | moderate MOD | | large MOD | |
| --- | --- | --- | --- | --- | --- |
| trial size | frequency | one-stage | two-stage | one-stage | two-stage |
| small | low | 0.51 (-1.52, 2.94)  -0.18 (4.46) | 0.37 (-1.29, 2.05)  -0.32 (3.34) | -0.04 (-3.07, 3.01)  -0.73 (6.08) | -0.05 (-2.01, 1.86)  -0.75 (3.87) |
| moderate | low | 0.44 (-0.44, 1.34)  -0.25 (1.78) | 0.44 (-0.41, 1.31)  -0.25 (1.72) | -0.10 (-1.47, 1.11)  -0.79 (2.58) | -0.09 (-1.37, 1.08)  -0.78 (2.45) |
| small | frequent | 0.51 (-0.61, 1.63)  -0.18 (2.25) | 0.49 (-0.61, 1.58)  -0.20 (2.18) | 0.14 (-1.17, 1.46)  -0.55 (2.64) | 0.13 (-1.16, 1.42)  -0.56 (2.59) |
| moderate | frequent | 0.49 (-0.09, 1.05)  -0.20 (1.14) | 0.50 (-0.09, 1.06)  -0.20 (1.16) | 0.13 (-0.68, 0.89)  -0.57 (1.57) | 0.12 (-0.71, 0.93)  -0.57 (1.64) |
|  | | | | | |
| substantial $\tau^{2}$ | | moderate MOD | | large MOD | |
| trial size | frequency | one-stage | two-stage | one-stage | two-stage |
| small | low | 0.47 (-1.89, 3.09)  -0.22 (4.97) | 0.34 (-1.45, 2.11)  -0.35 (3.56) | -0.12 (-3.23, 2.86)  -0.82 (6.09) | -0.10 (-2.06, 1.83)  -0.79 (3.89) |
| moderate | low | 0.42 (-0.81, 1.54)  -0.28 (2.35) | 0.42 (-0.72, 1.48)  -0.28 (2.20) | -0.11 (-1.74, 1.33) -0.81 (3.07) | -0.10 (-1.58, 1.26)  -0.79 (2.83) |
| small | frequent | 0.48 (-0.84, 1.79)  -0.21 (2.63) | 0.46 (-0.80, 1.71)  -0.23 (2.51) | 0.06 (-1.57, 1.59)  -0.63 (3.17) | 0.05 (-1.48, 1.52)  -0.64 (3.00) |
| moderate | frequent | 0.46 (-0.55, 1.34)  -0.23 (1.89) | 0.47 (-0.55, 1.35)  -0.22 (1.90) | 0.10 (-1.14, 1.09)  -0.59 (2.24) | 0.10 (-1.17, 1.12)  -0.59 (2.30) |

Posterior mean and 95% CrI are provided in the greyed area, followed by bias and width of 95% CrI (in parenthesis) in the white area.

**Table S9. Posterior mean (95% CrI) and bias (width of 95% CrI) for log OR (old versus placebo)**

| small $\tau^{2}$ | | moderate MOD | | large MOD | |
| --- | --- | --- | --- | --- | --- |
| trial size | frequency | one-stage | two-stage | one-stage | two-stage |
| small | low | 0.11 (-1.94, 2.27)  -0.30 (4.21) | 0.06 (-1.49, 1.62)  -0.35 (3.11) | -0.55 (-3.73, 2.05)  -0.96 (5.79) | -0.38 (-2.14, 1.39)  -0.78 (3.53) |
| moderate | low | 0.07 (-0.79, 0.90)  -0.33 (1.69) | 0.08 (-0.75, 0.88)  -0.33 (1.63) | -0.49 (-1.88, 0.67)  -0.89 (2.55) | -0.46 (-1.72, 0.65)  -0.86 (2.38) |
| small | frequent | 0.18 (-0.84, 1.18)  -0.23 (2.02) | 0.17 (-0.82, 1.15)  -0.23 (1.96) | -0.25 (-1.53, 0.96)  -0.66 (2.49) | -0.24 (-1.48, 0.95)  -0.65 (2.42) |
| moderate | frequent | 0.16 (-0.40, 0.69)  -0.24 (1.09) | 0.17 (-0.40, 0.70)  -0.24 (1.11) | -0.23 (-1.00, 0.49)  -0.64 (1.49) | -0.24 (-1.03, 0.52)  -0.64 (1.55) |
|  | | | | | |
| substantial $\tau^{2}$ | | moderate MOD | | large MOD | |
| trial size | frequency | one-stage | two-stage | one-stage | two-stage |
| small | low | 0.06 (-2.32, 2.33)  -0.34 (4.65) | 0.01 (-1.65, 1.63)  -0.39 (3.29) | -0.61 (-4.22, 2.19)  -1.02 (6.40) | -0.40 (-2.20, 1.40)  -0.80 (3.60) |
| moderate | low | 0.03 (-1.21, 1.14)  -0.37 (2.35) | 0.04 (-1.09, 1.07)  -0.37 (2.16) | -0.55 (-2.19, 0.83) -0.96 (3.01) | -0.51 (-1.92, 0.78)  -0.91 (2.70) |
| small | frequent | 0.14 (-1.28, 1.36)  -0.27 (2.63) | 0.13 (-1.18, 1.29)  -0.27 (2.47) | -0.33 (-1.87, 1.06)  -0.73 (2.93) | -0.31 (-1.73, 1.03)  -0.72 (2.76) |
| moderate | frequent | 0.14 (-0.85, 0.98)  -0.26 (1.83) | 0.15 (-0.84, 0.99)  -0.25 (1.83) | -0.25 (-1.38, 0.68)  -0.66 (2.07) | -0.26 (-1.40, 0.71)  -0.66 (2.11) |

Posterior mean and 95% CrI are provided in the greyed area, followed by bias and width of 95% CrI (in parenthesis) in the white area.

| **Note on the bias of posterior mean of NMA log OR** |
| --- |

The NMA log OR between new and old intervention is the parameter of interest. It is obtained through the consistency equation:

$$\mu_{NO}=\mu_{NP}-\mu_{OP}$$

where $\mu_{NP}$ and $\mu_{OP}$ are the true basic parameters that correspond to the NMA log OR of new and old interventions against placebo, respectively. The estimated NMA log OR between new and old intervention, $\hat{\mu}_{NO}$, equals the truth and the bias, that is:

$$\hat{\mu}_{NO}=\hat{\mu}_{NP}-\hat{\mu}_{OP}$$

$=\left( \mu_{NP}+b_{NP} \right)-\left( \mu_{OP}+b_{OP} \right)$

| $=\left( \mu_{NP}-\mu_{OP} \right)+\left( b_{NP}-b_{OP} \right)$ | (1) |
| --- | --- |

$=\mu_{NO}+b_{NO}$

where $\hat{\mu}_{NP}$ and $\hat{\mu}_{OP}$ are the estimated NMA log OR of new and old intervention against placebo, respectively, whereas $b_{NP}$ and $b_{OP}$ are the correspoding biases: their difference determines the bias of $\hat{\mu}_{NO}$. The consistency equation for $b_{NO}$ (equation (1)) is implicit from the consistency equation for $\mu_{NO}$. Then, the following are also implicit:

- if $b_{NP}>b_{OP}$, then, $b_{NO}>0$;
- if $b_{NP}<b_{OP}$, then, $b_{NO}<0$;
- if $b_{NP}\approx b_{OP}$, then, then, $b_{NO}\approx0$.

The latter indicates that both $\hat{\mu}_{NP}$ and $\hat{\mu}_{OP}$ may have considerable bias; however, due to the consistency equation imposed on $\hat{\mu}_{NO}$ and its bias, $b_{NO}$ may be estimated to be low, if and only if, $b_{NP}$ and $b_{OP}$ are similar. Contrariwise, $b_{NP}$ may be considerably larger or smaller than $b_{OP}$, and consequently, $b_{NO}$ will be substantial (in absolute value), as well.

As a result, the low bias in $\hat{\theta}_{NO}$ under the two-stage approach may be the result of having similarly large bias in $\hat{\theta}_{NP}$ and $\hat{\theta}_{OP}$, whereas the comparatively larger bias in $\hat{\theta}_{NO}$ under the one-stage approach may result from having a larger or small bias in $\hat{\theta}_{NP}$ as compared to $\hat{\theta}_{OP}$. Therefore, presenting only the results on the functional parameters of interest may be misleading, if there is substantial bias in at least one of the basic parameters of the model, as the bias on the functional parameter may be cancelled out to a substantial extent through the consistency equation.

| **Covariance between the within-trial log OR and within-trial standard error** |
| --- |


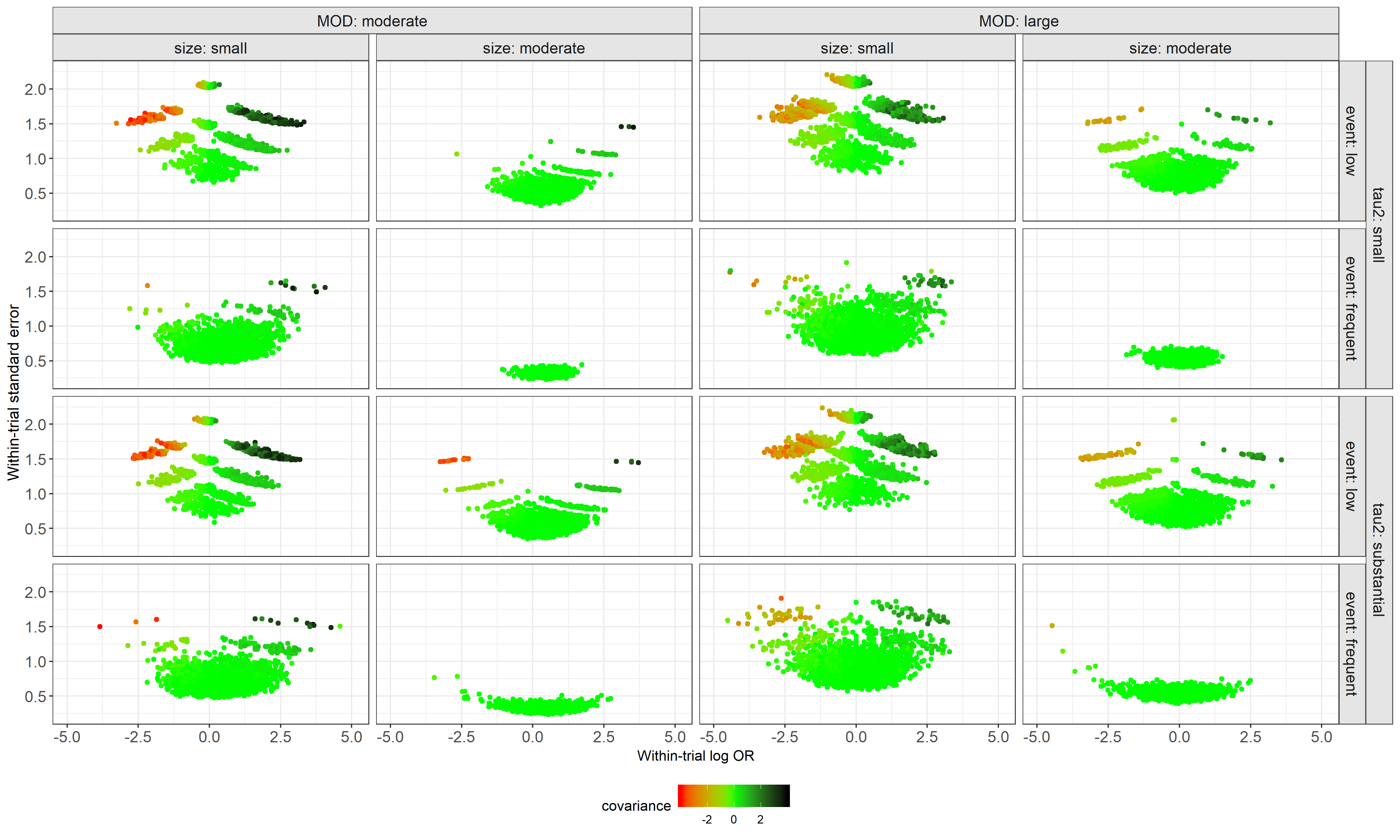


**Figure S1**. A panel of scatterplots on the within-trial standard error of log OR for ‘new intervention versus placebo’ (axis *y*) against the within-trial log OR for that comparison (axis *x*) for each simulation scenario. The colour key indicates the magnitude of covariance between the within-trial standard error of log OR and within-trial log OR for that comparison. MOD, missing outcome data; OR, odds ratio.


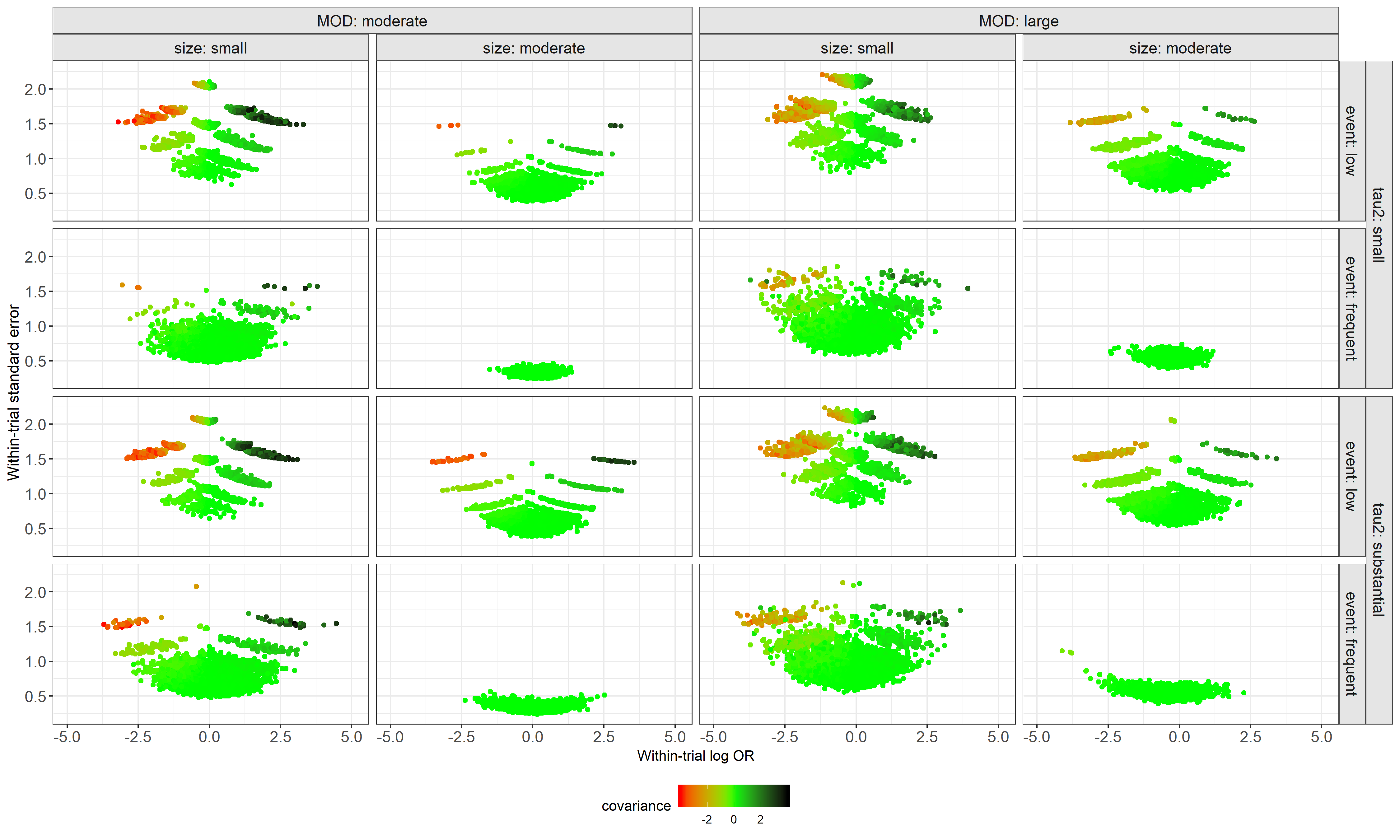


**Figure S2**. A panel of scatterplots on the within-trial standard error of log OR for ‘old intervention versus placebo’ (axis *y*) against the within-trial log OR for that comparison (axis *x*) for each simulation scenario. The colour key indicates the magnitude of covariance between the within-trial standard error of log OR and within-trial log OR for that comparison. MOD, missing outcome data; OR, odds ratio.

| **Low missing outcome data: bias in the posterior mean of NMA log OR** |
| --- |


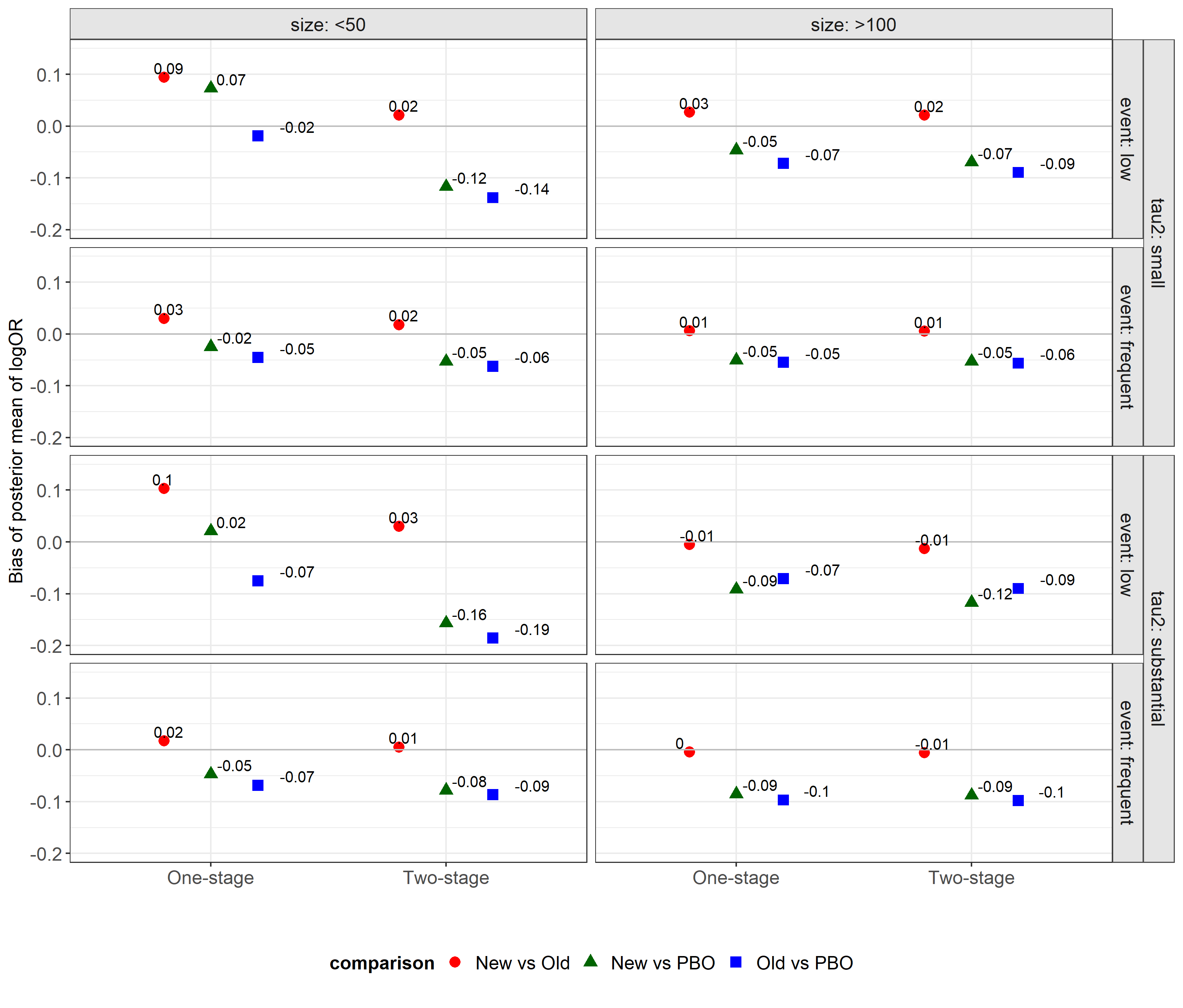


**Figure S3**. Dot plots on the bias of posterior mean of NMA log OR for all pairwise comparisons under one-stage and two-stage PM approaches while accounting for low missing outcome data, the size of trials (small, moderate), the event frequency (low, frequent) and the extent of $\tau^{2}$ (small, substantial).
